# IL-6 coaxes cellular dedifferentiation as a pro-regenerative intermediate that contributes to pericardial ADSC-induced cardiac repair

**DOI:** 10.1186/s13287-021-02675-1

**Published:** 2022-01-31

**Authors:** Hongtao Zhu, Xueqing Liu, Yuan Ding, Kezhe Tan, Wen Ni, Weili Ouyang, Jianfeng Tang, Xiaojun Ding, Jianfeng Zhao, Yingcai Hao, Zenghui Teng, Xiaoming Deng, Zhaoping Ding

**Affiliations:** 1 Department of Cardiology, The People’s Hospital of Danyang, Affiliated Danyang Hospital of Nantong University, West Xinmin Rd. 2, Danyang, 212300 China; 2 Department of Clinical Laboratory, Danyang Hospital for Chinese Traditional Medicine, Danyang, 212300 China; 3 Department of Anesthesiology and Critical Care, Changhai Hospital, Navy Medical University, Changhai Road 168, Shanghai, 200433 China; 4 Institute of Neuro and Sensory Physiology, Heinrich-Heine University of Düsseldorf, Moorenstr. 5, 40225 Düsseldorf, Germany; 5 Institute of Molecular Cardiology, Heinrich-Heine University of Düsseldorf, Moorenstr. 5, 40225 Düsseldorf, Germany

**Keywords:** Pericardial adipose stromal cells, Interleukin-6 (IL-6), Dedifferentiation, WT1, STAT3, Cardiac regeneration

## Abstract

**Background:**

Cellular dedifferentiation is a regenerative prerequisite that warrants cell cycle reentry and appropriate mitotic division during de novo formation of cardiomyocytes. In the light of our previous finding that expression of injury-responsive element, Wilms Tumor factor 1 (WT1), in pericardial adipose stromal cells (ADSC) conferred a compelling reparative activity with concomitant IL-6 upregulation, we then aim to unravel the mechanistic network that governs the process of regenerative dedifferentiation after ADSC-based therapy.

**Methods and results:**

WT1-expressing ADSC (eGFP:WT1) were irreversibly labeled in transgenic mice (WT1-iCre/Gt(ROSA)26Sor-eGFP) primed with myocardial infarction. EGFP:WT1 cells were enzymatically isolated from the pericardial adipose tissue and cytometrically purified (ADSC^gfp+^). Bulk RNA-seq revealed upregulation of cardiac-related genes and trophic factors in ADSC^gfp+^ subset, of which IL-6 was most abundant as compared to non-WT1 ADSC (ADSC^gfp−^). Injection of ADSC^gfp+^ subset into the infarcted hearts yielded striking structural repair and functional improvement in comparison to ADSC^gfp−^ subset. Notably, ADSC^gfp+^ injection triggered significant quantity of dedifferentiated cardiomyocytes recognized as round-sharp, marginalization of sarcomeric proteins, expression of molecular signature of non-myogenic genes (Vimentin, RunX1), and proliferative markers (Ki-67, Aurora B and pH3). In the cultured neonatal cardiomyocytes, spontaneous dedifferentiation was accelerated by adding tissue extracts from the ADSC-treated hearts, which was neutralized by IL-6 antibody. Genetical lack of IL-6 in ADSC dampened cardiac dedifferentiation and reparative activity.

**Conclusions:**

Taken collectively, our results revealed a previous unappreciated effect of IL-6 on cardiac dedifferentiation and regeneration. The finding, therefore, fulfills the promise of stem cell therapy and may represent an innovative strategy in the treatment of ischemic heart disease.

**Supplementary Information:**

The online version contains supplementary material available at 10.1186/s13287-021-02675-1.

## Background

Cardiac insufficient, or heart failure (HF), is the consequence of progressive loss of cardiomyocytes during pathologic damage, chronic stress, or aging [1]. The current therapeutical barrier of HF is the scarce replenishment of functional cardiomyocytes either by exogenous implantation or via inherent cardiogenesis [2], as adult mammalian cardiomyocytes are notoriously considered as terminally differentiated cells and incapable of reentering cell cycle to rebuild cardiac muscle. Although a very low but detectable rate of intrinsic renewal of cardiomyocytes has been found in heart [3], the mitotic activity in mammalian cardiomyocytes in late postnatal and adulthood remains a rare event and is clearly inadequate to prevent cardiac pathological deterioration after injury. As such, the quest for cardiomyogenic approaches in the adult mammalian heart remains a top priority for cardiovascular research and therapeutic interventional strategies to treat HF.

Although adult mammalian hearts do not naturally regenerate, cardiomyocytes are known to be able to adjust their morphology, cytoskeletal arrangement, metabolism, and contractility at the cellular level in response to multiple stimuli, indicating that they still harbor substantial plasticity in response to pathological stresses [4]. The morphological alterations, collectively termed as “dedifferentiation,” are characterized by loss of myofibril organization, return to immature status, and expression of embryonic genes and stem cell markers. The change in cytoarchitecture of cardiomyocytes has a direct impact on their ability to proliferate [5] and brings on a partial reminiscent of immature and replicating cardiomyocytes during cardiogenesis [6]. In the regenerative non-mammalians such as newt, for instance, dedifferentiation of internal stump cells in response to undefined signals is an essential step to form a mass of progenitor and pluripotent cells, known as the regeneration blastema, in the early limb regenerate [7]. In the context of myocardial repair in lower vertebrates, such as zebrafish, or neonatal mice in which where cardiac proliferation is fully functional in response to acute injury [6, 8], formation of new cardiomyocytes is found to begin with the initial step of cardiac dedifferentiation followed by mitotic cell division, suggesting that cellular dedifferentiation is a pro-regenerative prerequisite for tissue renewal [9]. Therefore, unraveling the regulatory mechanism(s) that governs dedifferentiation progression is fundamentally imperative to understand how cell cycles in adult mammalian cardiomyocyte are regulated and to develop therapeutical strategies that stimulate turnover of cardiomyocytes and promote cardiac regeneration.

Induction of cardiac dedifferential process involves multiple regulatory molecules, including microRNA, transcriptional factors, and paracrine cytokines [9]. Interleukin-6 (IL-6) family members are known to be important regulators that orchestrate dedifferential process during heart regeneration [10]. Oncostatin M (OSM) has been reported to be a master regulator of cardiomyocyte dedifferentiation in adult mammalian heart [11], and recently also to play a role in neonatal heart regeneration [12]. Cytokines of IL-6 family utilize the common IL-6 signal transducer, gp130, either homodimerization or heterodimerization, to stimulate multiple pathways including JAK/STAT (Janus kinase/signal transducers and activators of transcription), ERK (extracellular-signal-regulated kinase) and PKB (protein kinase B) [13]. Notably, in zebrafish after apical resection, a dynamic induction of IL-6 family-mediated Jak1/STAT3 signaling accompanied by cytokine production has been found in the regenerative progress [10], and inhibition of STAT3 in cardiomyocytes restrict the injury-induced proliferation and regeneration, indicating IL-6/STAT3 cascade is functionally important to cardiomyocyte regeneration, preferentially in the context of injury [14]. Moreover, the role of IL-6/STAT3 cascade in adult mammalian is also implicitly exemplified in a self-limited model of myocarditis in which compensatory proliferation of pre-existing cardiomyocytes was triggered by robust activation of STAT3 activity [15]. Thus, IL-6 signaling may act as one of the potent regulators of cardiac dedifferential process that has been proven as an essential step to initiate mitotic and proliferative activity in cardiomyocytes [9].

Interestingly, we have previously reported that IL-6 production was the most abundant in the cardiac transudate from the hearts received implementation of adipose stromal cells (ADSC) derived from the injury-responsive pericardial tissue [16]. This observation leads us to premise IL-6 signaling as an important dedifferential regulator that mediated the cardiac beneficial effects after ADSC transplantation, likely acting in a similar way seen in regenerative zebrafish hearts [14]. In this study, we identified IL-6 as one of the most upregulated cytokines in the injury-responsive ADSC expressing Wilms Tumor factor 1 (WT1) and demonstrated that mature cardiomyocytes were induced into dedifferential status in vivo and in vitro when treated with the tissue extracts prepared from ADSC-treated heart. Furthermore, we defined ADSC-derived IL-6 as an essential factor that triggers cardiac dedifferential step and confers functional benefits after ADSC-based therapy.

## Materials and methods

### Animal experiments

All the experiments were approved by the Institutional Animal Care and Use Committee at the Nantong University (NTU, S20200410-003) and conducted in accordance with standard operating guidelines for animal care. All animals (strain C57BL/6J, 20–25 g of body weight, 8–12 weeks of age) used in this study were bred at the Animal Center of NTU and fed with a standard chow diet and received tap water ad libitum.

The transgenic mice including WT1-iCre mice, Gt(ROSA)26Sor-EGFP reporter mice and IL-6 knockout mice (B6.129S2-IL6^tm1kopf^) were purchased from Jackson Laboratory (JAX, Bar Harbor, ME, USA). To generate tamoxifen-inducible expression of reporter gene (enhanced Green Fluorescent Protein, eGFP), WT1-iCre mice were crossed with ROSA26 strain. WT1-dependent expression of eGFP in pericardial tissue (eGFP:WT1) was induced by twice injections of 1 mg/day tamoxifen dissolved in 200 μl peanut oil (i.p.): one day pre- and 3 days post-myocardial infarction (MI), respectively. For the comparison, the transgenic littermates were used as controls and received tamoxifen injection using the same protocol.

Induction of MI in mice was performed as previously described [17]. In brief, mice were intubated, and ventilated mechanically at a rate of 180 strokes/min with tidal volume of 200 µl (Hugo Sachs, Germany). Anesthesia was maintained by continuous inhalation of gas mixture (25% O_2_ + 75% N_2_) containing 1.5% v/v isoflurane (Shandong Keyuan, China). All surgical procedures were performed on pre-warmed operation plate (37 °C) with the paws taped to an electrocardiogram (ECG) board (lead II, ADInstrument) to monitor ST segment elevation after cardiac ischemia. After dermal disinfection, the chest was opened with a lateral cut along the left side of the sternum, and the visible land-markers of the heart surface were identified. After the left artery descending (LAD) being identified, an 8–0 polypropylene suture (Prolene®, Ethicon) with a tapered needle was passed underneath LAD, the 1 mm from the tip of the left auricle. Successful occlusion of LAD was verified by both visual inspection under the microscope and immediate elevation of ST segment in the ECG registration. Cardiac ischemia was sustained for 60 min and cardiac perfusion was resumed by releasing the suture node on the LAD. Cardiac reperfusion was always confirmed by the immediate reddening of the heart surface and a transient downward shift of T-wave.

Intramyocardial injection was made by single injection of 20 µl of cell suspension (2 × 10^5^) or 20 µl of PBS by using U-100 insulin syringe with 30 G (0.30 mm × 8 mm, Micro-Fine™, BD) into the ventricular anterior wall (within the infarcted area) at the rate of 10 µl/min. After injection, animals were allowed to recover from injection procedure until the hemodynamics stabilized, and the chest was closed with one layer through the muscle and a second layer through the skin. Animals were weaned from ventilation and placed in a warm, oxygen-enriched environment until physical activity was fully recovered. Only mice that survived to the endpoint of experiments were included in data analysis.

### Assessment of cardiac function

Cardiac contractile function was assessed 5 weeks after MI surgery using ultra-miniature *P*–*V* loop catheters (Millar Instruments) and PVAN data analysis software [18]. Briefly, mice were intubated and anesthetized by inhalation of isoflurane (1.5% v/v) via mechanical ventilation and rectal temperature kept between 36.7 and 37.3 °C. A conductance 1.4 French microtip catheter (SPR-839) was inserted into the left ventricular cavity through a section in the middle of the right carotid artery (closed-chest). After at least 20 min stabilization of baseline measurement, the raw pressure (*P*) and relative volume unit (RVU) data were collected by the MPVS-Ultra® unit and then imported into the PVAN software platform for off-line analysis. A cuvette calibration was made to convert RVU into true volume units (µl) and thereby left ventricular *P*–*V* loop was reconstructed. Left ventricular developing pressure (LVDP) and its first-order deviates (d*P*/d*t*_max_ and d*P*/d*t*_min_) as well as ejection fraction (EF) were analyzed by LabChart 8 software (ADInstruments).

### Isolation and purification of murine pericardial ADSC

Pericardial adipose-derived stromal cells (ADSC) were isolated from the transgenic mice (WT1^CreERT±/eGFP+^ and IL-6^−/−^) and littermate controls 14 days after MI induction (injury-primed) as previously reported [19]. In brief, mice were sacrificed by overdose of isoflurane and the whole body was transiently immersed with 75% ethanol for about 1 min. Under a sterilized condition, the skin was removed, and the chest was then opened to expose the pericardiac tissue. The thoracis tissue including heart and part of lung was transferred to a sterilized, ice-cold PBS, and the pericardial tissue was carefully separated from the heart and thymus under stereomicroscope. After being washed twice with PBS, the pericardial adipose mass was minced into small pieces (about 1 × 1 × 1 mm^3^) and transferred to a 15-ml Falcon tube containing 2 ml of digestion solution (84 U/ml of collagenase II, Biochrom, Beijing, China). Digesting process was maintained at 37 °C for 20 min on shaker at a rate of 30 spm and thereafter stopped by adding 2 ml of FCS. After gently pipetting (10 X), cell suspension was spun down at 1000 rpm for 5 min to separate viable cells from cell debris and fat droplets. The cell pellet was re-suspended in a basic medium containing low sugar Dulbecco's modified Eagle's medium (DMEM, sugar = 1000 mg/ml, Sigma) supplemented with 30% FCS (sigma), penicillin (100 U/ml), streptomycin (0.1 mg/ml), and glutamine (1 mM). The derive ADSC were then seeded in a density of 2 × 10^3^/cm^2^ to T75 culture flask and incubated at 37 °C with 5% CO_2_. Non-adherent cells were removed 24 h after initial plating and the resulting adherent stromal fraction was then termed pericardial ADSC. The primarily isolated ADSC were cultivated at 37 °C with 5% CO_2_ for about 3–5 days until sub-confluence and further passaged.

The isolated ADSC were mixture of both eGFP:WT1 and non-eGFP:WT1 subpopulations. To obtain the pure progeny of WT1-expressing cells (eGFP:WT1) in which expression of eGFP have been implemented upon WT1 activation in response to tissue damage, we used the first passage of ADSC derived from WT1^CreERT±/eGFP+^ mice as mentioned above and separated by fluorescence-activated cell sorting (FACS, Canto™II, BD), resulting two subpopulations: eGFP:WT1 positive (ADSC^gfp+^) and eGFP:WT1 negative (ADSC^gfp−^) subsets. After FACS sorting, two types of cells were cultivated in the same condition until 3rd passage (P3) for cell transplantation experiments.

To characterize and compare the biological features of ADSC^gfp+^ and ADSC^gfp−^ subsets, cells were incubated with fluorochrome-conjugated antibodies (CD44, CD45, CD90, CD73, CD105 and Flk-1, listed antibodies is shown in Additional file 1) at room temperature for 10 min. After three times washing with PBS containing 10% FCS, cells were ready to be analyzed using a FACS Canto™II flow cytometer running CellQuest software (BD). For detecting intracellular and nuclear antigens, we employed immunocytochemical technique (ICC). In brief, cells were seeded on sterilized glass coverslips (24 × 24 mm) in 6-wall plate and cultured for 3 days until 80% sub-confluence. The cover slips were then fixed with 1% paraformaldehyde and permeabilized with 0.01% Triton for 10 min. Primary antibodies including monoclonal anti-WT1 (C-19), isl-1, GATA-4, nkx2.5, Tbx18 and polyclonal anti cardiac Troponin T (cTnT, 1:100) were added on the slips at dilution of 1:100 and incubated at 4 °C overnight. The slips were then washed for 3 times with 1% NGS-PBS buffer and incubated with secondary antibody (Cy3-conjugated goat IgG, 1:100) at room temperature for additional 60 min. Nuclei were counterstained with 4′,6-Diamidino-2-phenylindole (DAPI, Sigma) and the slips were mounted onto glass slides with Prolong Gold® (Life Science). All visualizations were digitalized by using a fluorescence microscopy (Olympus MX61) with software (CellSence®) or phase-contrast microscope using a digital camera (Olympus, UC30).

### Determination of the thickness of the left ventricular wall and the quantity of cardiomyocytes

Wall thickness of the anterior wall was used as a major anatomic measure to compare the effectiveness of structural repair in mice 35 days post-injection (dpi). In detail, after functional assessment, hearts were removed from the thorax and immediately placed into an ice-cold PBS chamber. Thereafter, both left and right ventricular chambers were carefully filled with liquid Tissue-Tek® and the hearts were properly placed in a vertical orientation within tissue block. Successive cryosections were made until the mid-ventricular level that corresponded the sites of injection. Sections (10 µm) were stained with Hematoxylin and Eosin (H&E) or Sirius Red and embedded with DPX mounting medium (Sigma-Aldrich) to illustrate the anatomic structure. All sections were photographed using a digital camera as abovementioned. The thickness of the left anterior wall was then manually derived from the average of 5 independent measure points of the radial distances between endocardial and epicardial plant in 3 successive sections (interval = 300 µm).

For quantitative analysis of cardiomyocytes, the adjacent cryosection to H&E staining was subjected to immunohistochemical staining (see below) of cardiac marker (cTnT) to identify cardiomyocytes. The total fluorescent intensity that corresponded to the number of cardiomyocytes was derived from the integration of pixels within the detection frame placed manually within the infarcted area received PBS- or cell injection and presented as arbitrary unit (a.u).

### Immunohistochemistry

For immunological analysis, heart sections were prepared at initial (1 or 5 dpi) and endpoint stages (35 dpi) as indicated in individual figures. Cryosection (7 μm) of raw heart tissue was fixed with 4% paraformaldehyde for 10 min at room temperature and blocked with 5% normal goat serum (NGS) for 1 h at room temperature. The primary antibodies (indicated in Additional file 1) were diluted in PBS with 1.0% NGS, and immunoreaction was ensued by overnight incubation at 4 °C. After 3 × washing with PBS, fluorochrome-conjugated secondary antibodies were added at dilution of 1:400 (TRITC conjugated) or 1:200 (FITC conjugated) and incubated at room temperature for 2 h. Cell nuclei were counterstained with DAPI and then slides were mounted with Prolong™ Gold (Thomas Fisher). Fluorescent images were acquired using a digital camera mounted on a microscope as described above.

### RNA-seq and bioinformatic analysis

The RNA sequencing (RNA-seq) analysis was commercially commissioned to Beyotime Biotech (Shanghai, China) and performed as previously described [20]. In brief, total RNA was isolated from the cultured ADSC^gfp−^ and ADSC^gfp+^ cells at passage 3 by using RNeasy mini kit (Qiagen, Germany) according to the manufacturer's instructions (*n* = 3 in each group). Purified RNA samples (500 ng) were used for library construction and sequenced on the Illumina Hiseq PE150 to acquire a paired-end read (150 bp). The differentially expressed transcripts (DET) were computed with *DESeq2* package (version 1.20.0) supplied by R software (version 3.4.4) via a likelihood ratio test implemented in *DESeq* function. Data were then subjected to functional enrichment analysis by STRING software (version 10.5). Hypergeometric tests were used to identify enriched terms and GO terms were analyzed and sorted when FDR < 10^–4^.

### Gene expressions and IL-6 concentration

After cytometric separation upon eGFP expression, total RNA was extracted from both ADSC^gfp+^ and ADSC^gfp−^ cells by using RNeasy mini kit as described above in RNA-seq experiments and converted to cDNA with a first-strand cDNA synthesis kit (Invitrogen, USA) according to the manufacturer's instruction. Quantitative real-time PCR (RT-PCR) was performed in StepOne PCR apparatus (Applied Biosystems) and relative gene expression of each targeted gene was normalized to GAPDH and calculated using 2^delta^ Ct value methodology. All RT-PCR assays were performed in duplicate. Primers for Taqman qPCR were commercially purchased (Thermo Fisher) and listed as following: WT1: Mm01337048_m1; Nkx2.5: Mm01309813_s1; GATA4: Mm01310448_m1; TBX5: Mm00803518_m1; Tnnt2: Mm00441920_m1, Postn: Mm01284913_g1; IL-6: Mm00446190_m1; HGF: Mm01135185_m1; IGF-1: Mm00439560_m1; Tgf-b1: Mm01178820_m1 and GAPDH: Mm99999915_g1.

The concentration of IL-6 was assessed by Quantikine ELISA kit (R&D Systems™, Shanghai, China) according to manufacturer instructions.

### Preparation of cardiac extracts and cultivation of neonatal cardiomyocytes

Cardiac extracts were made from the hearts of 3-dpi mice: either received ADSC^gfp+^ injection or same volume of PBS control (see above). In detail, the middle part of the anterior wall was excised and immediately snap-frozen in liquid nitrogen. On the day of extraction, the cardiac tissue was weighted, thawed in 12 ml of DMEM medium containing cocktail of protease inhibitors (1%, Sigma-Aldrich). The tissue mass was ground with an electronic tissue homogenizer for 2 min and sonicated for additional 30 s. Then, the homogenate was first spun at 2000 g for 20 min to remove cell debris and the supernatant was further spun at 100,000 g for 60 min. After aspirating the insoluble lipid layer, the supernatant was collected and sterilized through a 0.45 µm-filter (Sartorius, Germany). The protein content was estimated with a BCA protein assay kit (Pierce) and all aliquots were stored at − 80 °C for cell culture experiments.

Neonatal cardiomyocytes were isolated from murine newborns (P1-2). In brief, the neonatal mice were sacrificed by decapitation and disinfected by transiently immerging the body in 70% ethanol. After opening the thorax, the hearts were collected and washed with ice-cold PBS. The ventricular parts of 3 hearts were minced into small pieces (1 × 1 × 1 mm^3^) and transferred into a 15-ml Falcon tube containing 2 ml of digestion solution (120 U/ml of collagenase II, Biochrom, Beijing, China). After enzymatic dissection at 37 °C for 20 min, digestive process was terminated by adding 2 ml of FCS. After centrifugation, supernatant was removed and cell pellet was re-suspended in DMEM medium supplemented with 10% FCS, penicillin (100 U/ml), streptomycin (0.1 mg/ml), and preliminarily seeded into a T75 culture flask. Two hours later, the non-adherent cells were collected and transferred to 6-well plates containing sterilized coverslips. The pre-plating protocol ensures a significant enrichment of cardiomyocyte fraction in culture.

To induce dedifferentiation of neonatal cardiomyocytes, 200 µl of cardiac extracts (either form PBS or ADSC^gfp+^ injected hearts) and anti-IL-6 monoclonal antibody (200 µg/ml, MP5-20F3, BioCell) were added to the culture medium (10%, v/v) 24 h after initial cultivation. Medium and extracts were changed daily, and after 5-day cultivation, the cells were fixed and stained with antibody anti-cardiac α-actinin (ACT, 1:400) using the protocol described above. All experiments were performed in duplicates and images of cardiomyocytes were photographed from at least 10 randomly selected area (2 × 5 fields in duplicates) and, according to organization of sarcomeric structures, the ratio of mature cardiomyocytes (mat-CM) in relation to dedifferentiated cardiomyocytes (dedi-CM) was calculated.

### Statistical analysis

Data were presented as mean ± standard deviation. A student *t* test with Welch’s correction was applied to compare the expression of surface markers and transcriptional factors, and IL-6 concentration between ADSC^gfp−^ and ADSC^gfp+^ subsets. All reparative and hemodynamic parameters as well as number of dedifferentiation were compared with one-way analysis of variance (ANOVA). Differences were considered significant at *p* < 0.05. The Prism software package (version 8.0) was used for the statistical analysis.

## Results

### WT1-expressing ADSC showed favorable cardiac repair over non-WT1 cells

**Fig. 1:**
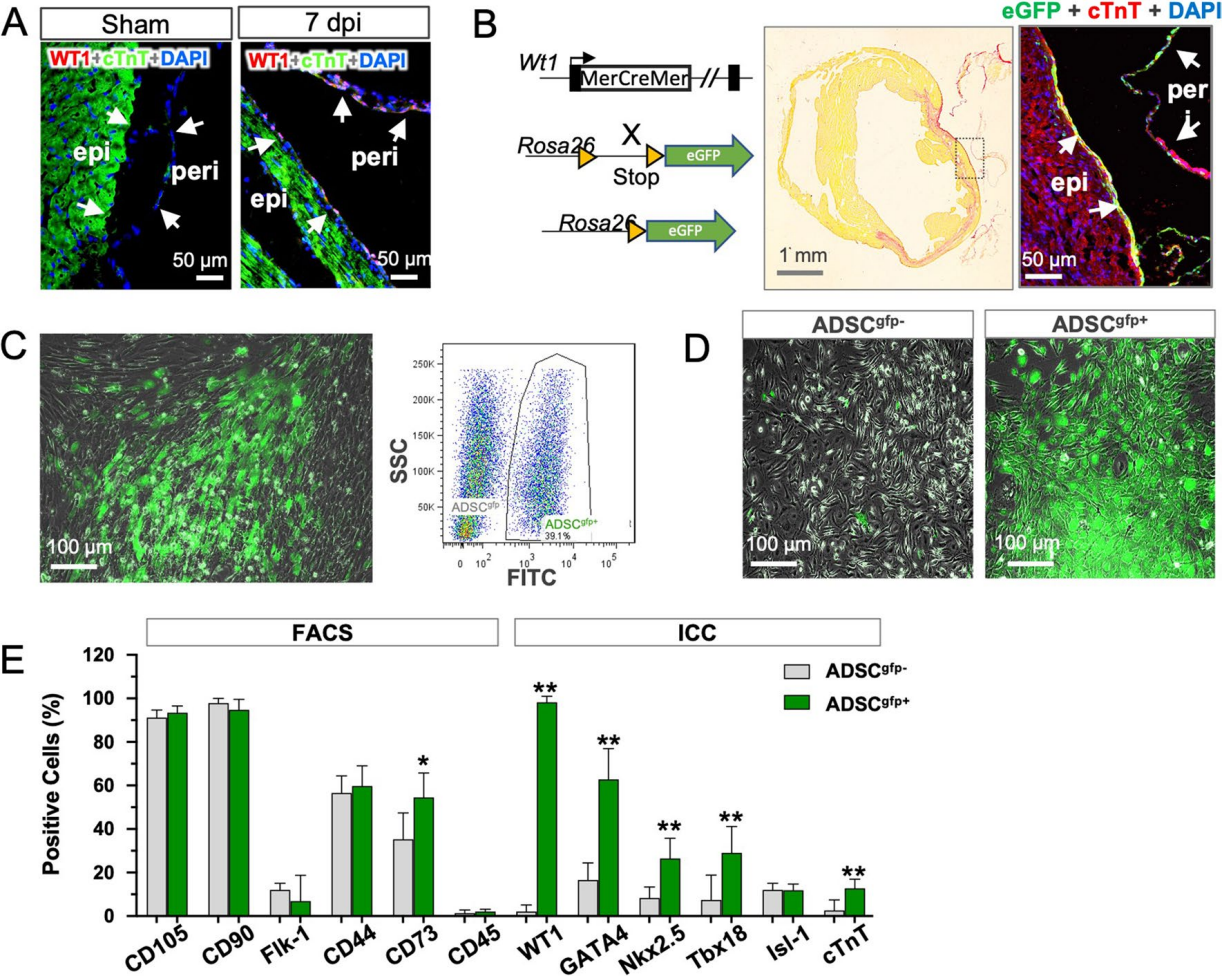
Isolation and purification of eGFP:WT1 cells from pericardial adipose tissue. **A** Expression of an injury-responsive element, Wilms Tumor factor 1 (WT1), which was almost undetectable in the uninjured heart (Sham), was found to be induced after myocardial infarction (MI, 7 days post in injury, dpi) in both epicardial layer (epi) and pericardial tissue (peri). **B** Schematic drawing shows the crossbreeding of mouse strain in which a reporter gene (enhanced green fluorescent protein, eGFP) was driven by WT1 expression (left) and irreversibly labeled the WT1 expressing cells in epicardial and pericardial tissue (right). **C** EGFP:WT1 cells from the pericardial adipose stromal tissue (ADSC^gfp+^) was isolated and cultured (left), and segregated form the non-WT cells (ADSC^gfp−^) by fluorescence-activated cell sorting (FACS, right). **D** Both ADSC^gfp−^ and ADSC^gfp+^ cells were cultured and passaged with relatively high purity (> 98%, *n* = 5). **E** FACS and immunocytochemistry (ICC) staining revealed both subpopulations (ADSC^gfp+^ and ADSC^gfp−^) shared similar epitope markers for mesenchymal stem cells, but ADSC^gfp+^ subset showed significant upregulation of cardiac-related genes (*n* = 5 in each group). *p < 0.05; **p < 0.01

We have previously reported WT1 expression in pericardial ADSC as an injury-responsive element that, once activated, boosted reparative activity in the rat model of infarction [16]. In the present experiments, we took the advantage of mouse model of Cre-transgenic mice in which activation of WT1 expression was irreversibly labeled with reporter gene (eGFP:WT1). As shown in Fig. 1A, immunodetection of WT1 expression confirmed positive staining of WT1 in the injured hearts (MI, 5 days), showing both epicardial and pericardial locations, while almost no WT1 expression was found in the uninjured hearts (Sham). The murine results were in line with previously reported data and verified WT1 reactivation as a reinstatement of developmental program in adult tissue in response to injury [21]. In the transgenic strain, eGFP:WT1 positive cells were readily detectable in both the epicardial layer and epicardial tissue 2 weeks after infarction (Fig. 1B, 32.2% vs. 24.2%, respectively), while eGFP expression was almost absent in both the epicardial and the pericardial cells in sham animals (data not shown), To obtain the pericardial origin of eGFP:WT1 cells, we enzymatically dissected the mass of pericardial adipose tissue and, after primary cultivation, eGFP negative population (ADSC^gfp−^) and eGFP-positive ADSC (ADSC^gfp+^) was separated by FACS sorting (Fig. 1C), leading significant enrichment of ADSC^gfp+^ cells with a purity up to 98.91% (Fig. 1D). Phenotypic characterization of the two subpopulations (ADSC^gfp+^ vs. ADSC^gfp−^) by flow cytometry (FACS, surface markers) of by immunocytochemistry (ICC, intracellular and nuclear markers) revealed both sunsets equally expressed markers for mesenchymal cells, with one exception of CD73 that was slightly upregulated ADSC^gfp+^ subset (*p* < 0.05), but both subsets were absent from hematopoietic marker (CD45). Note that ADSC^gfp+^ subset uniformly retained high proportion of WT1 expression (98.2%) after isolation and cultivation in vitro, while ADSC^gfp−^ subset was eventually negative (Fig. 1E). Interestingly, ADSC^gfp+^ subset also exhibited cardiac potential as evidenced by upregulation of several key cardiac transcriptional factors and cardiac protein (Fig. 1E) and development of spontaneous beating activity in culture dish (see Additional file 2), which coherently confirmed our previously reported results in rat model [16].

**Fig. 2:**
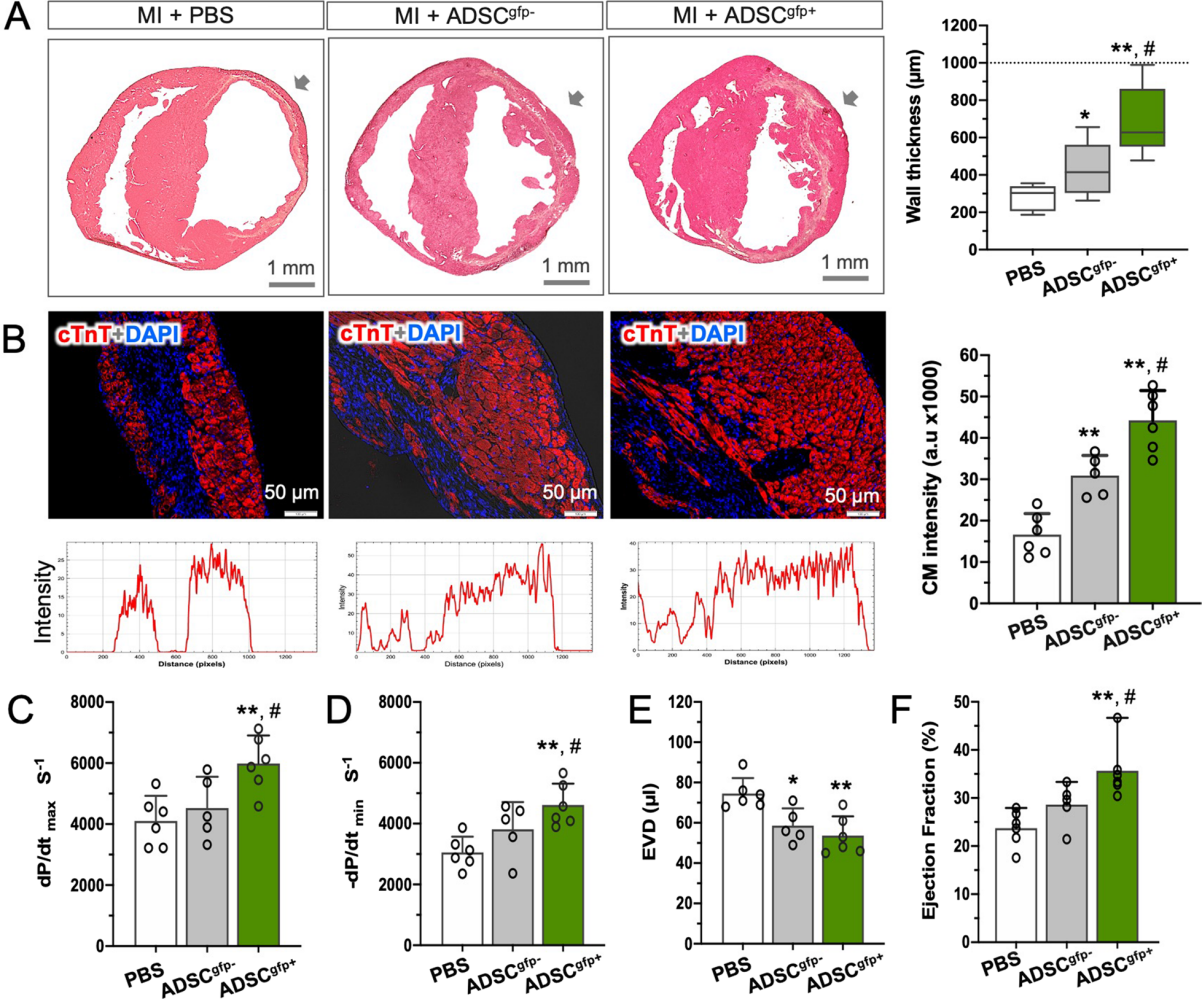
ADSC^gfp+^ subpopulation was more potent in cardiac structural repair and functional improvement. **A** Representative H&E staining at the mid-ventricular level showed a thin, fibrotic ventricular wall after myocardial infarction in the PBS-treated hearts (MI + PBS, *n* = 6), and injection of either ADSC^gfp−^ (MI + ADSC^gfp−^, *n* = 5), or ADSC^gfp+^ (MI + ADSC^gfp+^, *n* = 6) cells mended the infarcted wall at the site of injection (arrows). Note that the ADSC^gfp+^ subset yielded more pronounced structural repair. Dotted line: wall thickness of non-infarcted hearts. **B** Identification of cardiomyocytes (CM) by immunostaining for cardiac Troponin T (cTnT) revealed almost doubled intensity of red fluorescence (CM) by ADSC^gfp−^ injection (*n* = 5) and tripled in the ADSC^gfp+^ injected hearts (*n* = 6) in comparison to PBS controls (*n* = 6). **C**–**F** Cardiac contractile (d*P*/d*t*_max_) and relaxing (d*P*/d*t*_min_) properties, end-diastolic volume (EDV) and ejection fraction were derived from Millar catheter from three groups (*n* = 6, 5 and 6, respectively). Note that, injection of ADSC^gfp+^ cells significantly prevented the deterioration of cardiac function in PBS-treated MI hearts, while injection of ADSC^gfp−^ cells only yielded tendentious but insignificant improvement of most cardiac parameters. *p < 0.05; **p < 0.01 compared to PBS control. ^#^*p* < 0.05 compared to ADSC^gfp−^-treated hearts

In order to head-to-head compare the pro-regenerative activity of the two populations, we injected the same amount of two types of cells into the ventricular free wall of the infarcted hearts and compared the structural and functional benefits 5 week after treatment (35 dpi). In the mid-ventricular section where cells were applied, we found that, in comparison to PBS control (MI + PBS), both subsets of ADSC significantly yielded thickening of the infarcted wall (Fig. 2A, *p* < 0.01) in which more cardiomyocytes were detected (Fig. 2B, cTnT positive, *p* < 0.01). Notably, the reparative effect was more pronounced in the mice that were treated with ADSC^gfp+^(MI + ADSC^gfp+^) as compared to ADSC^gfp−^-treated animals (MI + ADSC^gfp−^, *p* < 0.05), demonstrating that, in line with our previously reported data [16], activation of WT1 expression in ADSC was associated with a distinct ability to mend the damaged hearts. Furthermore, the structural benefits were readily translated into functional improvement assessed by Millar *P*–*V* catheter (Additional file 3, Additional file 4). Ischemic injury let to a compromised contraction (d*P*/d*t*_max_) and relaxation (d*P*/d*t*_min_) properties with enlarged end-diastolic and end-systolic volume. The mice received ADSC^gfp+^ injection exhibited a significant alleviation of cardiac contractile function, indicated by improvement of d*P*/d*t*_max_ and d*P*/d*t*_min_ (Fig. 2C, D, *p* < 0.01), prevention of dilatative cardiomyopathy (EDV, Fig. 2E, *p* < 0.01), and restoration of ejection fraction (Fig. 2F, *p* < 0.01). While the ADSC^gfp−^-treated mice showed a tendency of improvement in relation to PBS controls, the effectiveness was minor and statistically insignificant, with one exception of EDV (Fig. 2E, *p* < 0.05). Thus, WT1-expressing cells showed a unique ability to repair the damaged hearts by building up a thick infarcted wall with significantly more cardiomyocytes and to improve contractile function of the compromised hearts.

### IL-6 mediated the reparative activity of ADSC^gfp+^ subset

**Fig. 3:**
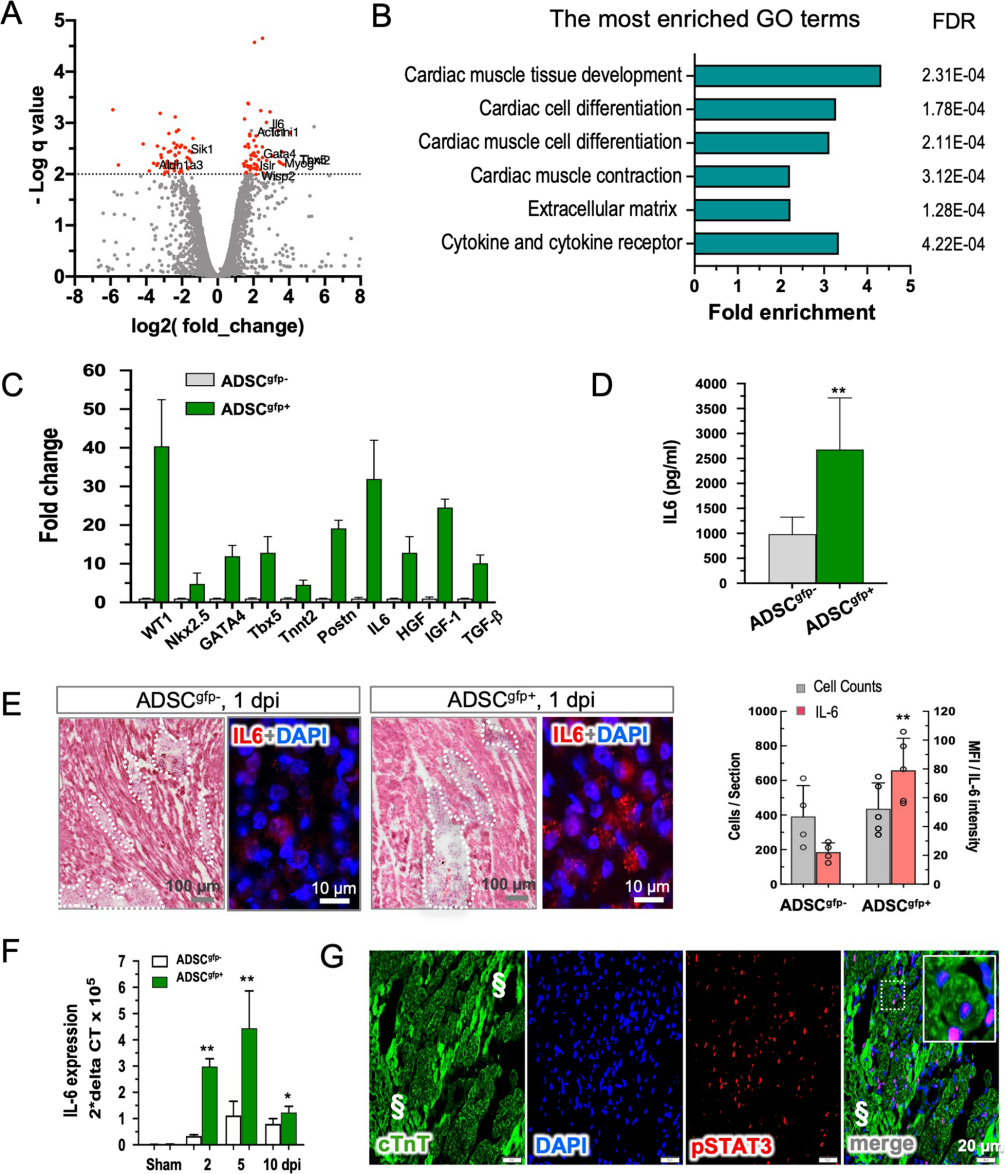
Identification of IL-6 as the most abundant cytokines secreted by ADSC^gfp+^. **A** Volcano plotting of bulk RNA-seq data illustrated a panel of differential expression genes between ADSC^gfp+^ (*n* = 3) and ADSC^gfp−^ subpopulations (*n* = 3). **B** Gene ontology analysis categorized the up-regulated genes into GO-enriched terms related to cardiac differentiation and cardiogenesis, extracellular matrix formation and cytokine release. **C** RT-PCR analysis confirmed the upregulation of the selected genes from RNA-seq analysis. The mean expression level of ADSC^gfp−^ was set as factor 1 (*n* = 5). Note striking up-regulation of WT1 and IL-6 expression in ADSC^gfp+^ subpopulation (*n* = 6). **D** Secretion of IL-6 was found significantly abundant in culture medium of ADSC^gfp+^ subset in comparison to ADSC^gfp−^ subset (ELISA, *n* = 4, 5). **E** H&E staining was used to define the interstitial location of the injected cells in the host tissue, and immunodetection to compare the expression level of IL-6 in the injected cells at the early stage (1 dpi, left). Quantitative analysis revealed that both types of cells (ADSC^gfp+^ vs. ADSC^gfp+^) yielded identical cardiac retention (*p* > 0.05), but significantly high mean fluorescence intensity (MFI) of IL-6 staining in the ADSC^gfp+^ subset (*p* < 0.01, *n* = 5 and 5, right). **F** RT-PCR detection of IL-6 expression in the cardiac tissue demonstrated a transient increase in IL-6 expression, peaking at 5 dpi. Remarkably, in the ADSC^gfp+^-injected hearts (*n* = 5) IL-6 expression was found significantly higher at all time points in comparison to ADSC^gfp−^-treated hearts (*n* = 4). **G** An important downstream nuclear target of IL-6, pSTAT3, was found to be activated exclusively in the viable cardiomyocytes after the treatment with ADSC^gfp+^ subset. § indicated dead myocardium (characterized by stark autofluorescent background in immunostaining). *p < 0.05; **p < 0.01

We asked ourselves what made ADSC^gfp+^ subset distinct from ADSC^gfp−^ subpopulation in the context of reparative capacity. We then performed RNA deep sequence and compared mRNA profile of the two types of cells. Analysis of differentially expressed genes identified 277 significantly up-regulated and 209 down-regulated genes that mostly related to cardiac muscle tissue development (GO:0048738), cardiac cell differentiation (GO:0035051) and cardiac muscle cell contraction (GO:0060048) (Fig. 3A, B). Quantitative RT-PCR analysis of the selected genes further showed 5–10 fold up-regulation of cardiac genes in ADSC^gfp+^ subset in relation to ADSC^gfp−^ subset (Fig. 3C), suggesting that WT1 expression in ADSC increased myogenic potential as we previously reported [16]. Interestingly, gene ontology also revealed enrichment of cytokine and cytokine receptor interaction (GO0005125) in ADSC^gfp+^ subpopulation (Fig. 3B). Quantitative RT-PCR testified upregulation of several trophic factors for tissue hemostasis and repair after injury, and among the cytokines examined, IL-6 was the most highly abundant at the mRNA level (32-fold in relation to ADSC^gfp−^ subset, Fig. 3C, *p* < 0.01) and the protein level in the culture medium detected by ELISA (2.5-fold, Fig. 3D, *p* < 0.01). Therefore, in addition to enhanced cardiac differential potential, ADSC^gfp+^ subset constituted a unique feature of IL-6 upregulation that likely linked to the reparative activity after being transplanted into the damaged hearts.

In the view of our previous results showing the most abundant IL-6 in the pericardial fluid after ADSC treatment [16], we reasoned that IL-6 signaling may act as one of the reparative factors that mediated the beneficial effects after ADSC-based therapy. To this end, we examined the IL-6 expression at the early time point soon after transplantation (1 dpi). In the hearts received both types of cells (ADSC^gfp−^ and ADSC^gfp+^), the injected cells were found to nestle in the interstitial space in small clusters (30–200 counts) and were morphologically distinguishable from the host cells in H&E sections as well as in DAPI staining (dense nuclei, see Additional file 5). The cell counts in both groups of hearts were almost identical, indicating equal cardiac retention regardless of cell types (Fig. 3E, *p* > 0.05). However, IL-6 expression in ADSC^gfp+^ cells was significantly abundant in comparison to ADSC^gfp−^ subset (Fig. 3E, *p* < 0.01), while the host cells exhibited a weak level of IL-6 expression (see Additional file 5), suggesting the implanted ADSC^gfp+^ subset were major source of IL-6 production at the early time point of injury (Fig. 3E). In the necrotic tissue, fluorescent tracking of the implanted cells by immunostaining was always hampered by technical obstacles of stark background of autofluorescence due to necrotic cell debris. Therefore, we performed quantitative RT-PCR to compare IL-6 expression between two groups and found that injection of either type of cells (ADSC^gfp−^ and ADSC^gfp+^) increased IL-6 expression readily from 2 until 10 dpi, peaking at 5 dpi in the cardiac tissue. Notably, ADSC^gfp+^ yielded more pronounced increase in IL-6 expression than in the ADSC^gfp−^ hearts, being manifested in the initial stage (6-fold at 2 dpi and 4-fold at 5 dpi, Fig. 3F, *p* < 0.01), and less significant at 10 dpi (30%, *p* < 0.05). Although tissue IL-6 expression was a mixture of both host cells and implanted cells, ADSC^gfp+^ subset was found to be far more potent than ADSC^gfp−^ subset at all the time points, suggesting injection of ADSC^gfp+^ subset was able to create a transient, IL-6-enriched niche that likely favors cardiac repair. To explore whether the elevated IL-6 expression was functionally relevant in cardiac tissue, we examined the downstream nuclear target of IL-6 signal transduction. Intriguingly, phosphorated STAT3 (pSTAT3) was found in the viable, but not in dead cardiomyocytes (indicated by § in Fig. 3G). By quantitation, we identified a frequency of 234 ± 34 pSTAT3 positive cardiomyocytes per section in ADSC^gfp+^ hearts, while only 82 ± 26 in ADSC^gfp−^ hearts (*p* < 0.01). Therefore, IL-6 and its downstream cascade are biologically active and likely mediate to ADSC^gfp+^-induced reparative activity in the damaged myocardium.

### IL-6 mediated dedifferentiation of cardiomyocytes in vivo and in vitro

**Fig. 4:**
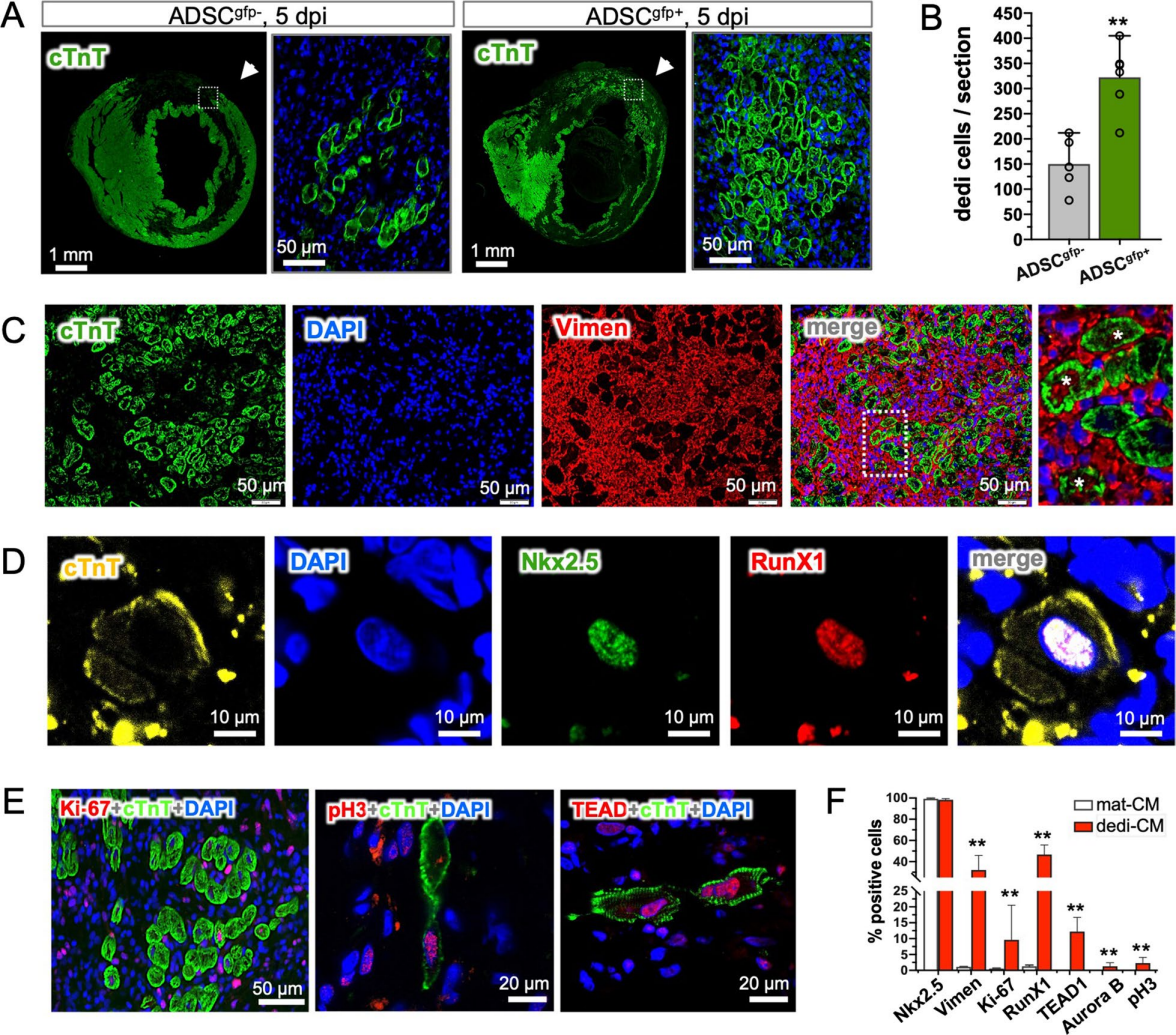
Characterization of ADSC^gfp+^-induced cardiomyocyte dedifferentiation. **A** Immunostaining with cardiac troponin T (cTnT) of the heart Sects. (5 dpi) illustrated endocardial and sub-epicardial location of surviving cardiomyocytes, while a few cTnT positive cells within the infarcted myocardium in the ADSC^gfp−^-treated hearts (left panel). In the ADSC^gfp+^-treated hearts (right panel), however, a significant amount of small, round shape of cTnT-positive cells were found within the infarct area, locating mainly at the site of injection (arrow). These cells situated within the infarcted area in a cluster pattern and typically exhibited marginalization of cTnT (dedifferentiation). **B** In each section of the ADSC^gfp+^-injected hearts, about 300 dedifferentiated (dedi) cells were detected (*n* = 6), while only half dedi cells were found in ADSC^gfp−^-treated hearts (*n* = 5). **C** The partial dedi-CM displayed intercellular expression of Vimentin (Vimen, indicated by asterisk). **D** The dedi cells retained the expression of cardiac fingerprint (Nkx2.5) and concomitantly stem cells marker (RunX1). **E** Representative immunostaining showed the dedi cells expressed ki-67 as a marker of cell cycle reentry, pH3 as mitotic activity and TEAD1 as downstream activation of YAP signaling. **F** Quantitative analysis of the ADSC^gfp+^-treated hearts (*n* = 8) revealed all dedi cardiomyocytes (dedi-CM) retained cardiac marker, partially gained mesenchymal properties and, in small fraction, proliferative activity, while those markers were almost absent in mature cardiomyocytes (mat-CM). **p < 0.01 compared to the ADSC^gfp−^-injected hearts in **B** and *p* < 0.01 compared to mat-CM in **F**

Given that the activation of STAT3 by one of the IL-6 family members, Oncostatin M, for instance, was able to initiate dedifferential progress in mature cardiomyocytes [11], we quantitatively analyzed the dedifferential process in the ADSC-treated hearts in early time point (5 dpi) when cell debris in the infarcted area has been resolved. Stained with cTnT, dedifferentiated cardiomyocytes (dedi-CM) were recognized by the unique feature of sarcomeric disassembly and marginalization of cardiac proteins [22]. While surviving cardiomyocytes were mainly found in the endocardial and subepicardial territories, the dedi-CM were found spatially within the infarcted area (Fig. 4A). Quantitatively, in the hearts treated with ADSC^gfp+^ subset, a significant number of round-shape dedi-CM were found, largely within the anterior wall in which ADSC^gfp+^ subset was applied (arrow in Fig. 4A), while the ADSC^gfp−^-treated hearts presented only spare, handful dedi-CM, locating only in the margin zone of survived myocardium (Fig. 4A, B, *p* < 0.01). The results implicitly suggested that eGFP:WT1 cells were more effective and potent to induce the formation of dedi-CM than non-WT1 cells. Histological characterization further illustrated that most of dedi-CM retained cardiac property (Nkx 2.5, 98.3%, Fig. 4F), but some of them expressed mesenchymal protein (Vimen, 32.1%, Fig. 4C, F) and RunX1 (46.7%, Fig. 4D, F). Interestingly, small fraction of dedi-CM showed the activation of important markers of cell cycle and proliferation (Ki-67, 9.6%, Aurora B, 1.3%, pH3, 2.3%) as well as of YAP-associated downstream target (TEAD1, 12.2%) (Fig. 4E, F), while expressions of those markers were almost absent in mature cardiomyocytes (mat-CM) (Fig. F). The results suggested that, once formed, dedi-CM were able to reenter cell cycle and gained proliferative activity that may contribute to de novo formation of cardiomyocytes.

**Fig. 5:**
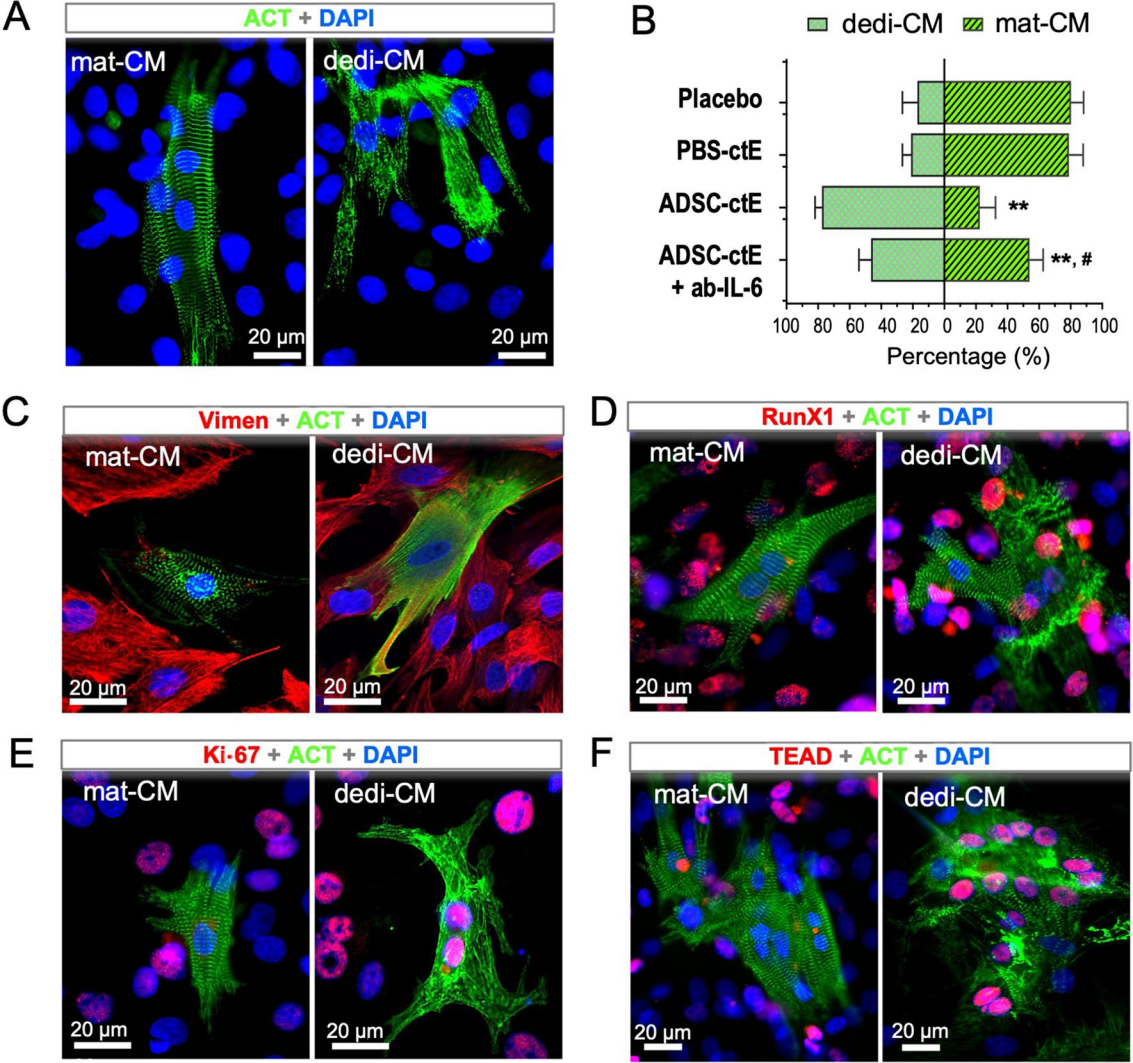
Obligatory role of IL-6 in the induction of cardiac dedifferentiation in isolated neonatal cardiomyocytes. **A** In cultured neonatal cardiomyocytes, existence of mature cardiomyocytes (mat-CM) and dedifferentiated cells (dedi-CM) was categorized by cardiac a-actinin (ACT) staining upon their distinct morphological appearance: the mat-CM showed a clear striation pattern, while the dedi-CM featured a sarcomeric assembly. **B** Spontaneous dedifferentiation in the cultured neonatal cardiomyocytes after 5-day cultivation was estimated at the rate of 20% in placebo controls (*n* = 5). Addition of tissue extracts from PBS control hearts (PBS-ctE) did not alter the dedifferential rate, but when cardiac tissue extracts from ADSC^gfp+^-treated hearts (ADSC-ctE, 10%, *n* = 5), was supplemented, the rate was accelerated up to 78%, leading to the ratio of both types of CM significantly shifted to dedi-CM dominating statue in the culture. The proportional shift of two populations was partially reverted by supplement of monoclonal antibody against IL-6 (ab-IL-6, *n* = 4). **C–F** While the mat-CM showed almost negative staining for mesenchymal cells (Vimen and RunX1), cell proliferation (Ki-67) and YAP-responsive target (TEAD1), the dedi-CM cells were stained positive for those markers, which was found likewise in vivo situations. **p < 0.01 compared to the PBS-ctE treated cells and ^#^*p* < 0.01 compared to ADSC-ctE treated cells

To explore the impact of IL-6 on myogenic dedifferentiation, we performed in vitro assay in primarily isolated neonatal cardiomyocytes. Short-term cultivation (5 days) of neonatal cardiomyocytes resulted in a mixture of mature cardiomyocytes (mat-CM, characterized with intact sarcomeric striations, Fig. 5A, left panel) and small fraction of spontaneously dedifferentiated cardiomyocytes (dedi-CM, featured by sarcomeric disassembly, Fig. 5A, right panel) at a ratio of 20% (dedi-CM vs. mat-CM, Fig. 5B). The percentage was not altered by adding tissue extracts of the PBS control hearts (PBS-ctE, 21.1%, *p* > 0.05), but, when the tissue extracts from the hearts that received ADSC^gfp+^ injection was added (ADSC-ctE, 10%), the ratio was shifted to a dedi-CM dominating phenotype (77.5%, *p* < 0.01). Notably, the ADSC-ctE-induced shift was partially reversed by supplement of monoclonal antibody against IL-6 (ab-IL-6, 46.4%, *p* < 0.01), indicating that IL-6 plays an essential role in the process of myogenic dedifferentiation (Fig. 5B). Furthermore, we found that, while the mat-CM stained almost negative for mesenchymal and proliferative markers, dedi-CM showed expression of key molecular signatures for dedifferential process (Vimen and RunX1) and proliferative activity (Ki-67 and TEAD1) (Fig. 5C–F), which was phenotypically found in vivo situation (Fig. 4C–E). Therefore, our results revealed an important role of ADSC-derived IL-6 in triggering cardiac dedifferentiation and proliferation.

### The obligatory role of IL-6 on cardiac dedifferentiation and repair in vivo

**Fig. 6:**
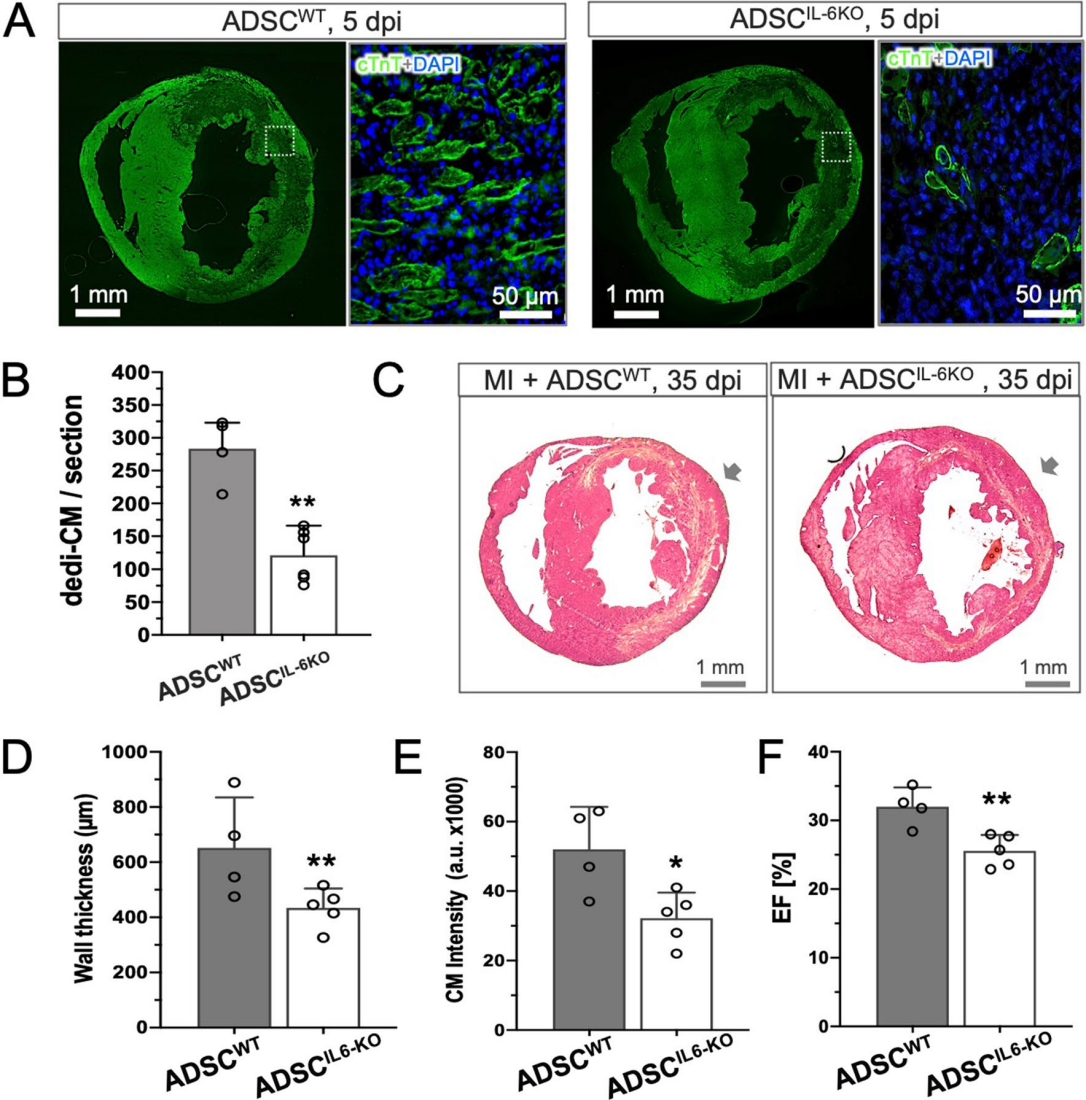
Reduced cardiac dedifferentiation and reparative activity as IL-6 expression was genetically lacking in ADSC. **A** Immunostaining of cardiac troponin T (cTnT) revealed significant formation of dedifferential cardiomyocytes typically exhibited marginalization of cTnT protein in hearts treated with either wildtype ADSC (ADSC^WT^) or ADSC lacking IL-6 production (ADSC^IL-6KO^). **B** Quantitative comparison illustrated that ADSC^IL-6KO^-injection (*n* = 6) yielded less than half counts of dedifferentiated cardiomyocytes (dedi-CM) found in the ADSC^WT^-treated hearts (*n* = 4). **C** Representative H&E staining of MI heart at the mid-ventricular level illustrated an increase in wall thickness after injection of wildtype ADSC (MI + ADSC^WT^, indicated by arrow), which was diminished as ADSC lacking IL-6 expression (ADSC^IL-6KO^). **D** Quantitative analysis showed ADSC^IL-6KO^ treatment (*n* = 4) created less increase in ventricular wall thickness in comparison to the ADSC^WT^ injected hearts (*n* = 4). **E** A similar tendency of cardiomyocytes intensity (CM intensity) to wall thickness was found between two groups (*n* = 4 and 5, respectively).** F** Improvement of cardiac ejection fraction (EF measured by Millar catheter) in mice treated with ADSC^WT^ (*n* = 5) was significantly diminished in the ADSC^IL-6KO^-treated mice (*n* = 5). *p < 0.05, **p < 0.01

**Fig. 7:**
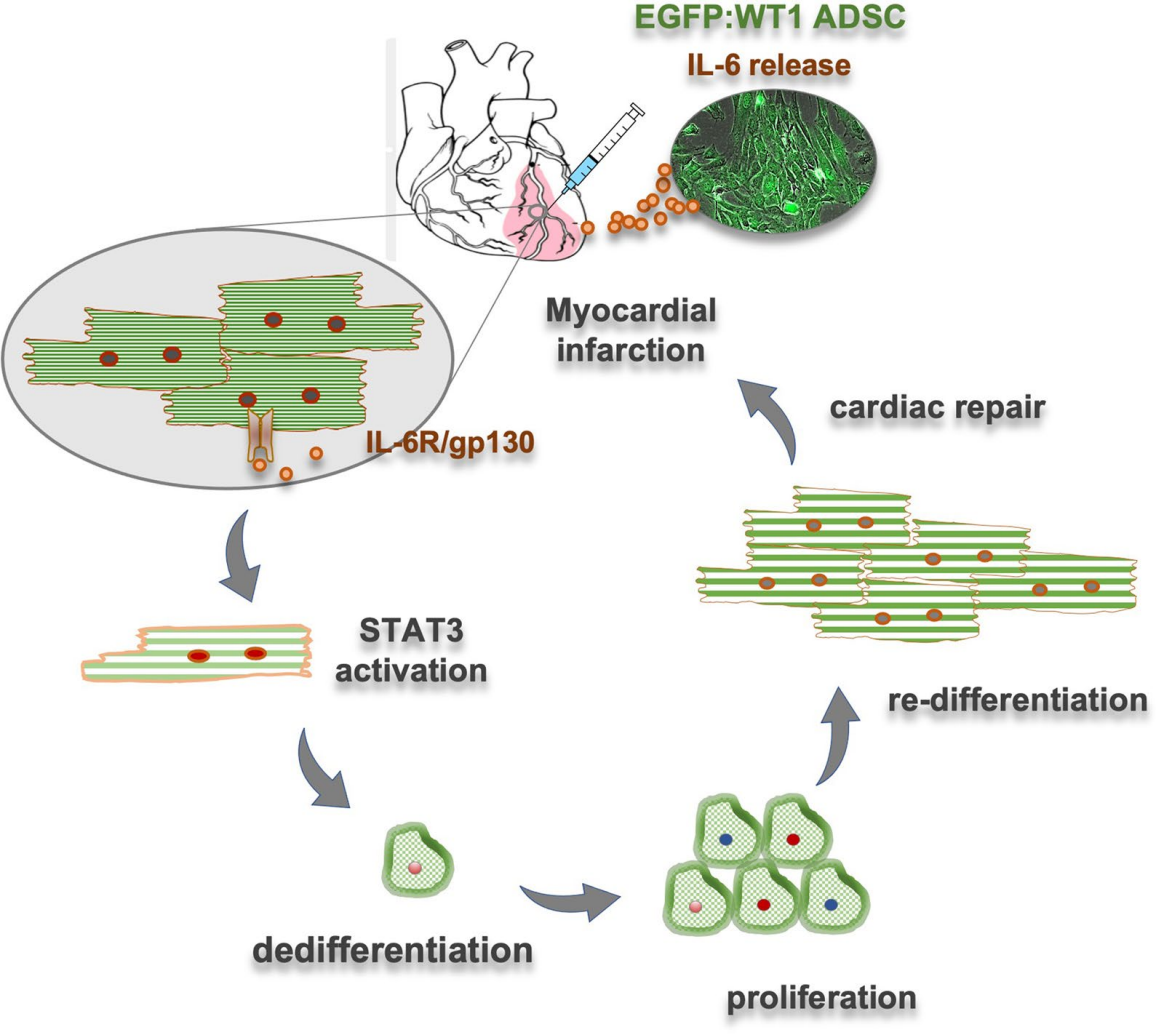
A proposed mechanistic loop of IL-6 mediated cardiac repair. Injection of WT1-expressing ADSC (eGFP:WT1 ADSC) into the damaged ventricular wall (myocardial infarction) led to an abundant production of interstitial IL-6 which subsequently activated its downstream nuclear target, STAT3, via IL-6/gp130 interaction on cell membrane. Activation of STAT3 in cardiomyocytes commenced a dedifferential process which warranted cardiac proliferation and regeneration of de novo cardiomyocytes for cardiac repair

To demonstrate the protective and pro-regenerative role of IL-6 in vivo, we isolated ADSC from IL-6 knockout mice (ADSC^IL-6KO^) and compared the reparative activity to wildtype ADSC (ASDC^WT^, unfractured). At the early phase of injection (5 dpi), cardiac dedifferentiation was detectable in the hearts received injection of both types of ADSC (Fig. 6A). Notably, significantly high quantity of dedi-CM was found in the hearts treated with ASDC^WT^ cells when compared to the ASDC^IL-6KO^-treated hearts (Fig. 6B, *p* < 0.01). Thirty-five days after injection, histological analysis illustrated that both types of cells yielded a thickening of the infarcted wall (Fig. 6C). While injection of ASDC^IL-6KO^ cells produced only minor effects, ADSC^WT^-injection resulted in significantly more pronounced increase in wall thickness at the site of injection (Fig. 6C, D, *p* < 0.01) and significant quantity of cardiomyocytes determined by immunostaining of cTnT (CM intensity, Fig. 6E, *p* < 0.05). Furthermore, the abovenamed structural benefits impacted also on cardiac contractile function: the compromised ejection fraction (EF) in ADSC^IL-6KO^-treated hearts was significantly alleviated in the ADSC^WT^-treated hearts (Fig. 6F, *p* < 0.01). Thus, these results pointed out an obligatory role of IL-6 in generating beneficial effects after ADSC-based therapy, likely via adequate formation of de novo cardiomyocytes via dedifferential, proliferative, re-differential process (Fig. 7).

## Discussion

Cellular dedifferentiation is a pro-regenerative step that warrants completion of mitotic division in adult mammalian cardiomyocytes. Here, we showed cardiac dedifferentiation as a pivotal step of cellular plasticity that associated with the reparative effects after stem cell-based therapy. Most importantly, we demonstrated that injury-responsive gene, WT1, was linked to upregulation of IL-6, which in turn mediated the regenerative activity of ADSC via promoting cardiac dedifferentiation at the onset of cardiac ischemia. While IL-6 is usually recognized as pro-inflammatory cytokine that causes cardiomyopathies, our results reveal a previously unappreciated role of IL-6 as a novel cardiac protective/pro-regenerative factor. This finding may strategically be used to empower current myocardial regenerative interventions in the treatment of HF.

### WT1 expression in ADSC promotes cardiac repair

Although cardiac stem cell-based therapies have been pursued in both basic and translational levels over 20 years, the heterogeneity of “stem cells” attributed to the efficiency of cardiac regeneration has been recently perceived [23]. Several studies have indicated that the reparative activity of tissue stem cells largely relies on the developmental origin [24, 25] and tissue environments where they locate [26]. In general, tissue mesenchymal stem cells (MSC) remain dormant during homeostasis and, in response to tissue injury, are activated by undefined factors to gain regenerative properties for tissue repair [27]. In this context, selection of therapeutically efficient stem cells is the most challenging, and so far, identification of such cell type has not yet been established [28]. In the stromal compartment of adipose tissue, multiple surface markers and transcriptional factors categorized ADSC into several sub-fractions that unevenly contribute to tissue homeostasis and repair. One of the interesting subpopulations was recently defined and termed as multilineage-differentiating stress-enduring (Muse) cells [29]. In the present study, we employed an injury-responsive expression of WT1 gene in the pericardial tissue as one of molecular hallmarks of recapitulation of embryonic program [30], as we have previously demonstrated that the injury-induced WT expression in ADSC exhibited an enhanced reparative activity after being implanted into ischemic rat hearts [16]. Although the Muse cell markers (i.e., SSEA3) was unchecked in the present study [31], we took the advantage of genetical approach that labeled irreversibly the progeny of WT-expressing cells by Cre-driven reporter gene expression (eGFP:WT1), and cytometrically purified the ADSC^gfp+^ from the pericardial tissue primed with ischemic injury (Fig. 1B). In line with our previously reported results in rat model, we found that the murine ADSC^gfp+^ subpopulation constituted a unique paracrine and transcriptional pattern linked to embryonic cardiogenesis, and yielded more pronounced beneficial effects after cardiac implantation (Fig. 2), suggesting that reparative activity was empowered in ADSC when WT1 expression was reactivated [16]. In this issue, this is also of interest in future study to compare the WT1-expressing cells to Muse cells in the context of surface markers and, more importantly, the reparative activities of the damaged tissues.

### IL-6 family/STAT3 cascade in tissue regeneration

Although ADSC^gfp+^ subpopulation demonstrated enhanced myogenic potentials (Figs. 1E, 2C) and developed spontaneous beating activity in vitro (Additional file 2), our previous cell-tracking experiments in rats have already proven that direct conversion and long-term engraftment of implanted cells minimally contributed to the beneficial effects of ADSC-based therapy [16]. In the present study, we focused on the paracrine activity of WT1-expressing cells that triggered endogenous plasticity, particularly by proliferation of pre-existing cardiomyocytes.

In an attempt to identify paracrine molecules/factors mediating the profound reparative activity, we performed RNA-deep sequence and DET analysis and found a broad-spectrum of upregulation of cardiac and trophic genes. Enriched expression of several cytokines and pleiotropic factors have been shown to contribute to cardiac protection and regeneration [32]. Interestingly, in line with our previous data showing IL-6 were most abundant in cardiac transudate after ADSC treatment [16], significant upregulation of IL-6 was found both in mRNA and in protein level (Fig. 3C, D), indicating IL-6 signaling may play an important role in ADSC-induced cardiac regenerative response that has been proposed recently [33]. IL-6 is a 26-kD secreted protein that belongs to the members of IL-6 family [34]. All IL-6 family members are involved in dimerization of the subunits of gp130 to initiate the downstream signaling by activation of Janus kinases and subsequent phosphorylation of cytoplasmic tyrosine residues, resulting in engagement of the tyrosine phosphatase Shp2 and activation of the downstream Ras/Erk and PI3K/Akt signaling cascades [13]. IL-6 is a pleiotropic cytokine in innate and adaptive immunity and is the most prominent example of a cytokine relevant to inflammatory diseases [34]. In cardiovascular research, the role of IL-6 signaling in mammals after myocardial infarction is highly debated. Inhibitory interventions of IL-6 signaling, using antibodies or small compounds, have been tested in clinical setting to prevent cardiac fibrosis and maladaptive remodeling in chronic HF patients [35]. However, accumulating evidence supports the notion that transient activation of IL-6/STAT3 signaling in the damaged tissue was cardiac protective and pro-regenerative both in zebrafish and mammalian hearts [36, 37]. IL-6 family/STAT3 signaling has been reported to be involved in myocardial regeneration mediated by the dedifferentiation and proliferation of cardiomyocytes, observed in the myocardium of neonatal rodents, as well as amphibians and fish [38], and lack of IL-6 production caused functional deterioration after myocardial infarction [39, 40]. In line with these reports, an engineered version of IL-6 (hyper IL-6), through GP130 to activate STAT3, was recently used successfully to promote central nervous system regeneration in adult mice [41]. However, it is interesting to note that the endogenous IL-6 production mainly relies on a sub-fraction of cardiac stromal cells [42] that, upon tissue injury, are activated and turned to be the major source of cardiac IL-6 production which drives to cardiac fibrosis, while the infiltrated immune cells (T cells/macrophages) contribute only minimally and transiently [43]. In such a scenario, the endogenous IL-6 production at the onset of cardiac ischemia is low and quantitatively ineffective in the induction of cardiac dedifferentiation that protects cardiomyocytes from ischemic damage. Thus, “timely” delivery of IL-6-bearing ADSC in the present study warranted adequate production of interstitial IL-6 (Fig. 3F, F, Additional file 5) which may subsequently induce the ischemic cardiomyocytes within the infarct area into a hibernating-like state. The hibernated/dedifferentiated cardiomyocytes require less oxygen supply and undergo a metabolic switch towards glycolysis that favors cardiac proliferation [44]. Therefore, it is likely that the impact of IL-6 on tissue homeostasis varies largely upon the tissue niche and the course of injury: it may act as a protector in early onset of damage-associated inflammation, while an accelerator that causes damage expansion due to unbridled inflammation and cardiac fibrosis, reflecting the “Janus face” of IL-6 in the regulation of tissue homeostasis and repair [45]. Furthermore, to substantiate the indispensable contribution of IL-6, we found that the injection of ADSC genetically lacking IL-6 production (IL-6 KO) brought about a comparably less cardiac benefits in relation to ADSC^WT^ cells (Fig. 6), underscoring the essential role of IL-6 mediating reparative activity after ADSC-based therapy.

### Cellular dedifferentiation as a pre-regenerative intermediate

With our accumulating knowledge about proliferation of pre-existing cardiomyocytes as a key origin of new cardiomyocytes, the cellular and molecular regulation of cardiac dedifferentiation during heart regeneration has been informatively pursued [9]. Cellular dedifferentiation is a process by which mature cells revert from their differentiated state back to a less-differentiated state to gain progenitor properties [3], characterized by a wide range of changes at the levels of gene expression, proteins, intuitive alternation of morphology and function, arguably more accurately called cellular plasticity [46]. In zebrafish and regenerative newborns, cellular dedifferentiation plays a crucial role in cardiac regeneration, which allows terminally differentiated cardiomyocytes to regain proliferative capacity, often notably by first reverting to stem cells or progenitor cells, followed by re-differentiation to functional cardiomyocytes [47]. In adult mammalian hearts, transient cardiac dedifferentiation is often viewed as cardiomyopathies, or simply a cellular plasticity in adaptation to stress and few of these studies has explored the proliferative capacity of mammalian dedifferentiated cardiomyocytes [48]. Apparently, the “dedifferentiation” of mammalian cardiomyocytes under unfavorable circumstances fails to reverse them back to the equivalent proliferative stage as observed during zebrafish heart regeneration. However, a proliferative capacity might be enhanced if the process could be experimentally manipulated through key regulators [3].

In the present experiments, we found that tissue content of IL-6/STAT3 signaling was an exquisite inducer of cardiomyocyte into a dedifferential status, likely acting in a same way as another IL-6 family members, OSM and IL-11 in mammalian heart [11] and zebrafish [10], suggesting that IL-6 family/STAT3 signaling is a conservative pathway to initiate dedifferential step under malignant stress [49]. The dedifferentiated cardiomyocytes exhibited a typical morphological alternation characterized by sarcomeric disorganization, relocation cardiac proteins towards the cell wall (marginalization), reduced cell–cell junctions and virtually increased cell mitosis markers (Aurora B and pH3 in Fig. 4). This notion was also supported by an in vitro study, in which cultured neonatal cardiomyocytes underwent dedifferentiation featured by loss of sarcomeric assembly and are concurrently positive for markers for cardiac progenitor and cell cycle reentry. Notably, the spontaneous dedifferential progression in cultured neonatal cardiomyocytes was accelerated by supplement of tissue extract of ADSC injected hearts, which was partially antagonized in the presence of anti-IL-6 antibody (Fig. 5), suggesting IL-6 as an inevitable inducer of cellular dedifferentiation. Therefore, it is likely that IL-6-induced adaptive dedifferentiation enforces cardiomyocytes into an intermediate, hibernating state for better survival in an unfavorable ischemic environment and, in meantime, small fraction of the dedifferentiated cells concomitantly enter cell cycle to proliferate (Fig. 7), a reminiscent of robust cardiac regeneration found in zebrafish and neonates [9].

Furthermore, activation of IL-6/STAT3 signaling also led to cell cycle re-entry in cardiomyocytes by YAP activation [12]. Interesting, YAP activation at default state was insufficient to promote cell growth in the absence of IL-6 co-stimulation [50], suggesting the adequate IL-6 production is needed to cooperatively force cardiomyocytes into active cell cycles. This notion was supported by our results showing re-expression of proliferative markers together with transcriptional enhancer factor 1 (TEAD1, Fig. 4E, F) as a downstream activation of YAP signaling in the dedifferentiated cardiomyocytes. On the other hand, while the present results have disclosed an important phenomenon of cardiac dedifferentiation that augmented by extracellular IL-6 production, we understand, however, that direct evidence linked the re-differentiation of the dedi-CM into de novo formation of cardiomyocytes that reconstitute the damaged part of the ventricular wall is still missing. One possible in vivo tracking of the dedi-CM relies on genetic tool that, once abovenamed non-cardiac markers within cardiomyocytes are induced, the fate decision of the dedi-CM could be followed with detection of reporter genes. These experiments needed to be carried out in future to address the important issue of re-differentiation of the dedi-CM into functional cardiomyocytes that contributes to cardiac repair in the damaged hearts.

## Conclusions

Although adult mammalian cardiomyocytes are considered to respond uncooperatively with abortive mitosis from checkpoint arrest, polyploidy with DNA synthesis without authentic cytokinesis, cellular dedifferentiation was still indispensably detected in the border zone of infarcted mouse hearts [51], suggestive of inherent cellular plasticity in response to injury. However, naturally occurring cardiac regeneration in vivo has proven to be far more nuanced and challenging. Given that cellular dedifferentiation–proliferation–redifferentiation are the regeneratively important steps toward cardiac regeneration [52], our results reveal an IL-6-mediated cardiac dedifferentiation that mechanistically contributes to the cardiac reparative activity after ADSC-based therapy. In the view that endogenous IL-6 content is at relatively low level at the early phase of MI, “timely” delivery of IL-6-bearing ADSC warrants an adequate interstitial IL-6 production that, by sequential activation of downstream nuclear target, STAT3, confers cardiac protection and dedifferentiation in the injured hearts. This finding, therefore, provides a fundamental path towards innovative strategies in developing therapeutic interventions that augment endogenous reparative activity in the treatment of HF.

## Supplementary Information

**Additional file 1.** List of antibodies used in histological and flow cytometric experiments.

**Additional file 2.** Spontaneous beating activity in ADSC^gfp+^ cells. First passage of ADSC^gfp+^ cells after FACS isolation was cultivation in DMEM medium with 8% FCS for about 10 days. A small fraction of eGFP positive cells (ADSC^gfp+^, lower left) developed spontaneous contraction in the culture dish. This video was recorded as a representative movie out of five independent experiments.

**Additional file 3.** Representative PV-loop recording derived by Millar catheter measurement. A 1.4 French microtip catheter was placed into the left ventricular cavity via right carotid artery and left ventricular developing pressure (*P*) and relative volume unit (RVU) data were simultaneously collected. Cardiac pressure volume-loop (PV-loop) was reconstructed in PVAN software platform. Note that right shift of the PV-loop (dilative cardiomyopathies) in PBS-treated hearts was alleviated by ADSC^gfp+^ treatment.

**Additional file 4.** Comparison of cardiac function assessed by Millar catheter. LVDP: left ventricular developing pressure; EDV: end-diastolic volume; ESV: end-systolic volume; SV: stroke volume; HR: heart rate; CO: cardiac output. *p* values were derived by one-way ANOVA.

**Additional file 5.** Stark IL-6 expression in the implanted ADSC^gfp+^ cells. In the early time point of ADSC^gfp+^ cell injection (1 day post-injection, 1 dpi), the implanted cells existed as a small cluster within the host myocardium (left, indicated by dense nuclei). Immunodetection of IL-6 revealed that, while the host cells expressed IL-6 at a weal level, the implanted cells showed strong fluorescent signal far above the autofluorescent background (right, *n* = 5).

## Data Availability

Raw data and the necessary detail can be provided by the corresponding authors under reasonable request.

## Abbreviations

ACT: Cardiac α-actinin
ADSC: Adipose-derived stromal cells
ADSC^gfp^^+^: EGFP:WT1 positive cells derived from pericardial adipose tissue
ADSC^gfp−^: EGFP:WT1 negative cells derived from pericardial adipose tissue
ctE: Cardiac tissue extracts
cTnT: Cardiac troponin T
dedi-CM: Dedifferentiated cardiomyocytes
dpi: Days post-infarction/injection of ADSC
eGFP:WT1: WT1-activated reporter gene (enhanced Green Fluorescent Protein)
IL-6: Interleukin 6
IL-6^KO^: IL-6 knockout
mat-CM: Mature/intact cardiomyocytes
MI: Myocardial infarction
STAT3: Signal transducer and activator of transcription 3
WT: Wild type
WT1: Wilms Tumor factor 1
