# Supplementary material for: IL-6 coaxes cellular dedifferentiation as a pro-regenerative intermediate that contributes to pericardial ADSC-induced cardiac repair
Source: Stem Cell Res Ther. 2022 Jan 31;13:44. doi: 10.1186/s13287-021-02675-1 (PMC8802508; doi:10.1186/s13287-021-02675-1)
Supplement: Supplementary file 1 — Additional file 1. List of antibodies used in histological and flow cytometric experiments. [file 13287_2021_2675_MOESM1_ESM.pptx]

## Slide 1
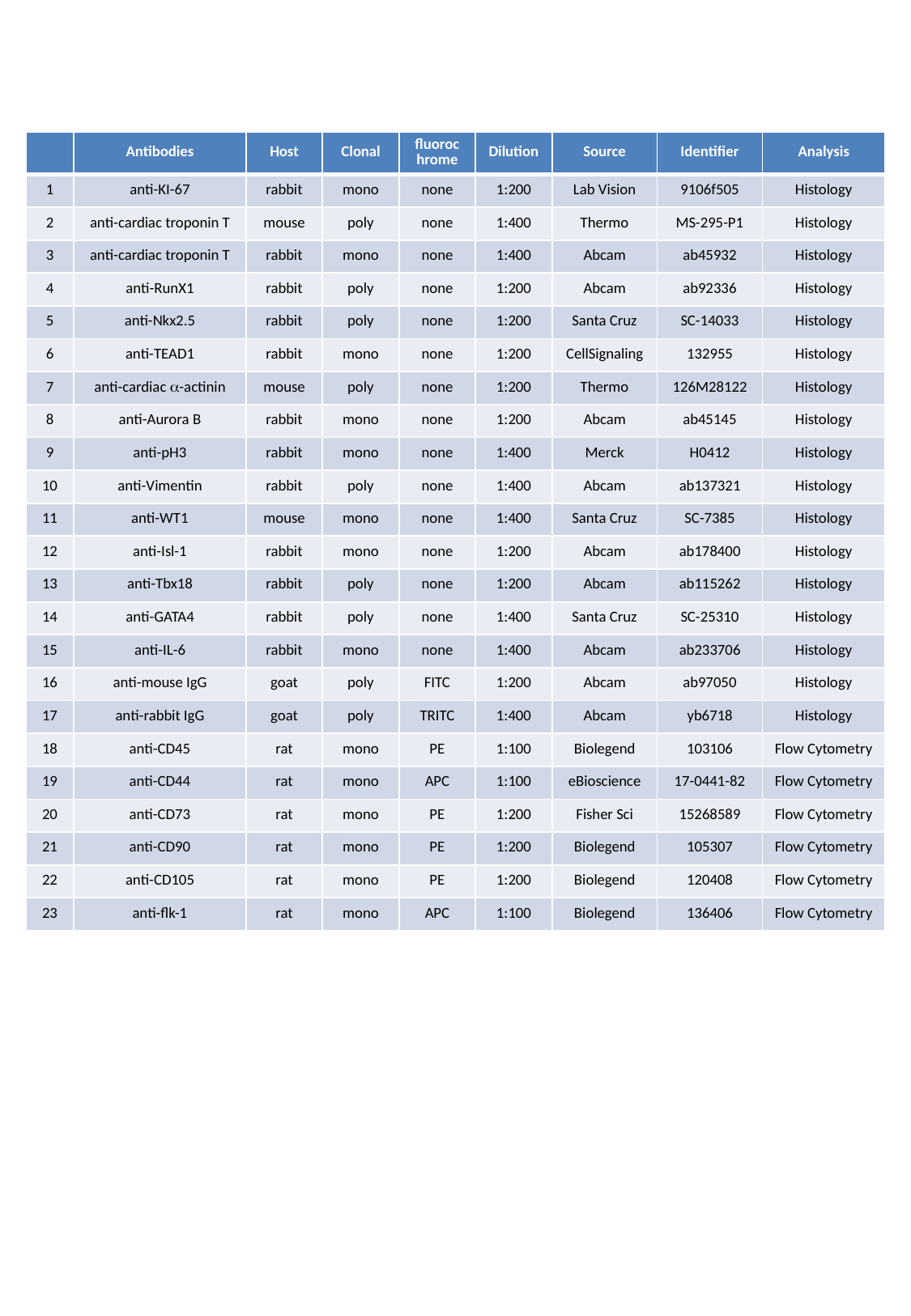

| | Antibodies | Host | Clonal | fluorochrome | Dilution | Source | Identifier | Analysis |
| --- | --- | --- | --- | --- | --- | --- | --- | --- |
| 1 | anti-KI-67 | rabbit | mono | none | 1:200 | Lab Vision | 9106f505 | Histology |
| 2 | anti-cardiac troponin T | mouse | poly | none | 1:400 | Thermo | MS-295-P1 | Histology |
| 3 | anti-cardiac troponin T | rabbit | mono | none | 1:400 | Abcam | ab45932 | Histology |
| 4 | anti-RunX1 | rabbit | poly | none | 1:200 | Abcam | ab92336 | Histology |
| 5 | anti-Nkx2.5 | rabbit | poly | none | 1:200 | Santa Cruz | SC-14033 | Histology |
| 6 | anti-TEAD1 | rabbit | mono | none | 1:200 | CellSignaling | 132955 | Histology |
| 7 | anti-cardiac a-actinin | mouse | poly | none | 1:200 | Thermo | 126M28122 | Histology |
| 8 | anti-Aurora B | rabbit | mono | none | 1:200 | Abcam | ab45145 | Histology |
| 9 | anti-pH3 | rabbit | mono | none | 1:400 | Merck | H0412 | Histology |
| 10 | anti-Vimentin | rabbit | poly | none | 1:400 | Abcam | ab137321 | Histology |
| 11 | anti-WT1 | mouse | mono | none | 1:400 | Santa Cruz | SC-7385 | Histology |
| 12 | anti-Isl-1 | rabbit | mono | none | 1:200 | Abcam | ab178400 | Histology |
| 13 | anti-Tbx18 | rabbit | poly | none | 1:200 | Abcam | ab115262 | Histology |
| 14 | anti-GATA4 | rabbit | poly | none | 1:400 | Santa Cruz | SC-25310 | Histology |
| 15 | anti-IL-6 | rabbit | mono | none | 1:400 | Abcam | ab233706 | Histology |
| 16 | anti-mouse IgG | goat | poly | FITC | 1:200 | Abcam | ab97050 | Histology |
| 17 | anti-rabbit IgG | goat | poly | TRITC | 1:400 | Abcam | yb6718 | Histology |
| 18 | anti-CD45 | rat | mono | PE | 1:100 | Biolegend | 103106 | Flow Cytometry |
| 19 | anti-CD44 | rat | mono | APC | 1:100 | eBioscience | 17-0441-82 | Flow Cytometry |
| 20 | anti-CD73 | rat | mono | PE | 1:200 | Fisher Sci | 15268589 | Flow Cytometry |
| 21 | anti-CD90 | rat | mono | PE | 1:200 | Biolegend | 105307 | Flow Cytometry |
| 22 | anti-CD105 | rat | mono | PE | 1:200 | Biolegend | 120408 | Flow Cytometry |
| 23 | anti-flk-1 | rat | mono | APC | 1:100 | Biolegend | 136406 | Flow Cytometry |
