# Supplementary figures and images for: IL-6 coaxes cellular dedifferentiation as a pro-regenerative intermediate that contributes to pericardial ADSC-induced cardiac repair
Source: Stem Cell Res Ther. 2022 Jan 31;13:44. doi: 10.1186/s13287-021-02675-1 (PMC8802508; doi:10.1186/s13287-021-02675-1)

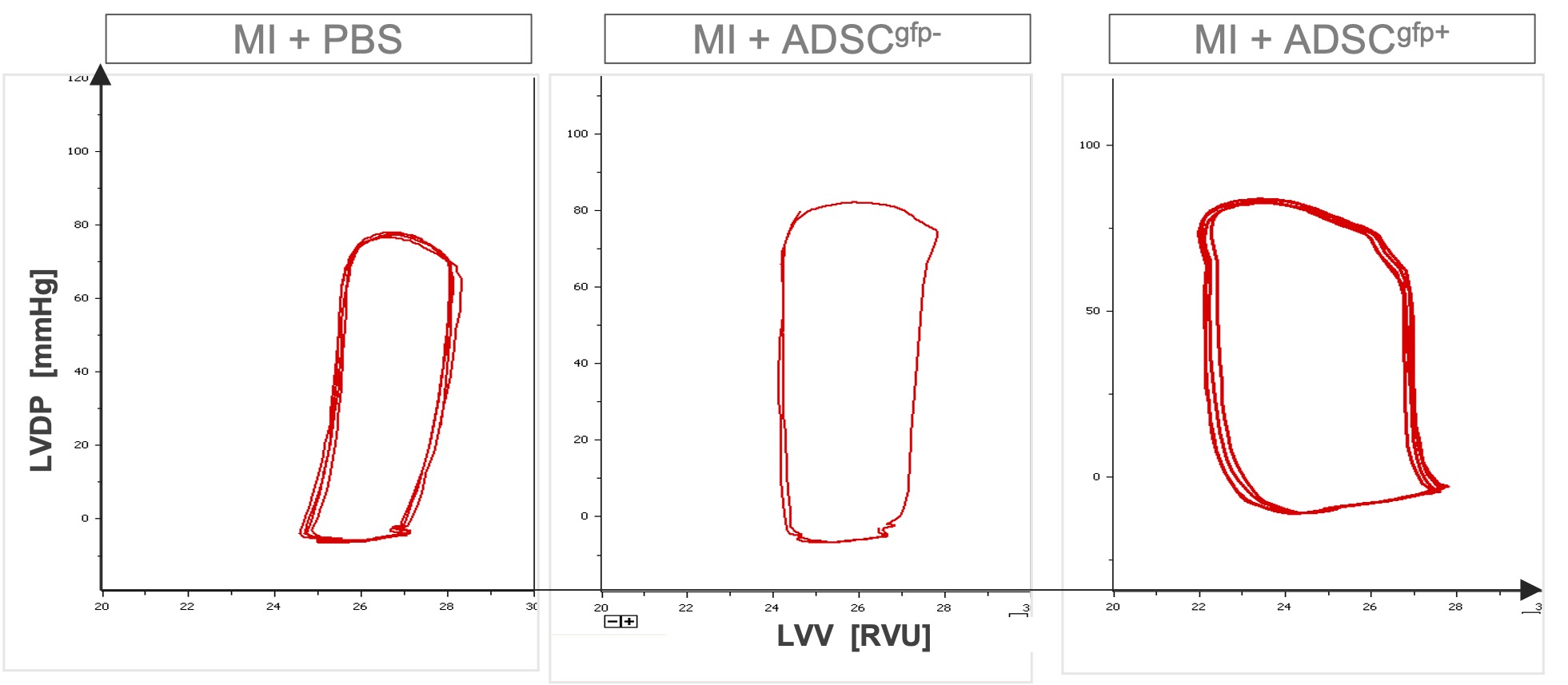

Supplement: Supplementary file 3 — Additional file 3. Representative PV-loop recording derived by Millar catheter measurement. A 1.4 French microtip catheter was placed into the left ventricular cavity via right carotid artery and left ventricular developing pressure (P) and relative volume unit (RVU) data were simultaneously collected. Cardiac pressure volume-loop (PV-loop) was reconstructed in PVAN software platform. Note that right shift of the PV-loop (dilative cardiomyopathies) in PBS-treated hearts was alleviated by ADSCgfp+ treatment. [file 13287_2021_2675_MOESM3_ESM.jpg]

## Slide 1
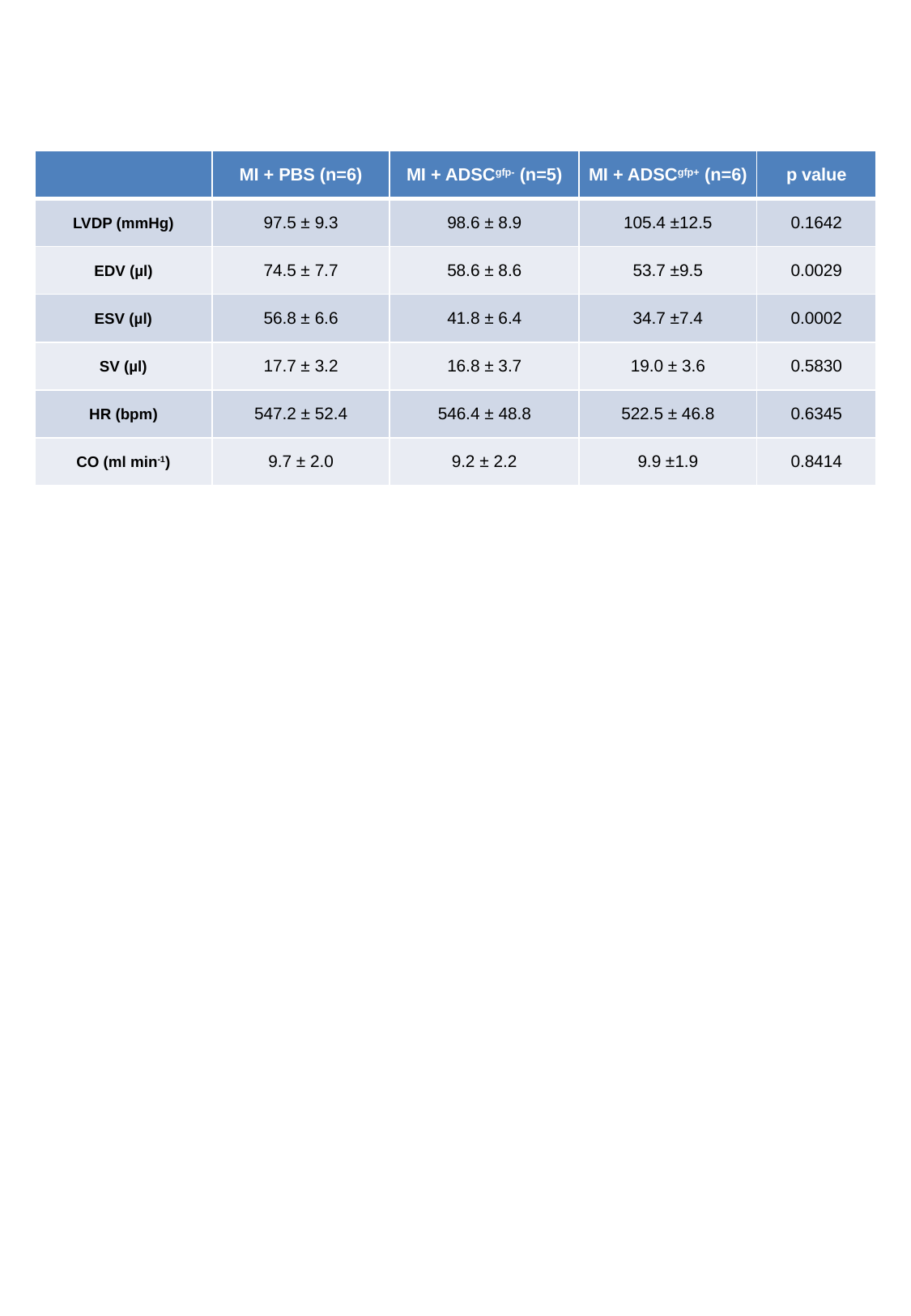

| | MI + PBS (n=6) | MI + ADSCgfp- (n=5) | MI + ADSCgfp+ (n=6) | p value |
| --- | --- | --- | --- | --- |
| LVDP (mmHg) | 97.5 ± 9.3 | 98.6 ± 8.9 | 105.4 ±12.5 | 0.1642 |
| EDV (µl) | 74.5 ± 7.7 | 58.6 ± 8.6 | 53.7 ±9.5 | 0.0029 |
| ESV (µl) | 56.8 ± 6.6 | 41.8 ± 6.4 | 34.7 ±7.4 | 0.0002 |
| SV (µl) | 17.7 ± 3.2 | 16.8 ± 3.7 | 19.0 ± 3.6 | 0.5830 |
| HR (bpm) | 547.2 ± 52.4 | 546.4 ± 48.8 | 522.5 ± 46.8 | 0.6345 |
| CO (ml min-1) | 9.7 ± 2.0 | 9.2 ± 2.2 | 9.9 ±1.9 | 0.8414 |

Supplement: Supplementary file 4 — Additional file 4. Comparison of cardiac function assessed by Millar catheter. LVDP: left ventricular developing pressure; EDV: end-diastolic volume; ESV: end-systolic volume; SV: stroke volume; HR: heart rate; CO: cardiac output. p values were derived by one-way ANOVA. [file 13287_2021_2675_MOESM4_ESM.pptx]

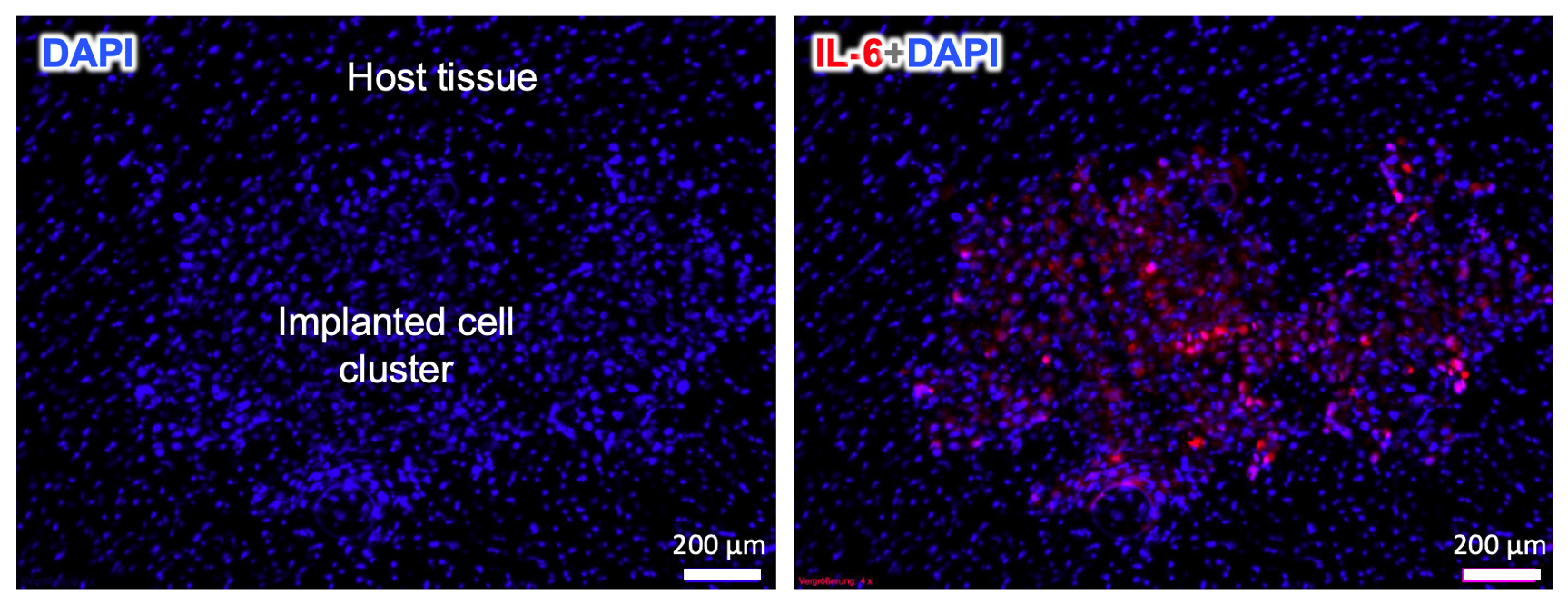

Supplement: Supplementary file 5 — Additional file 5. Stark IL-6 expression in the implanted ADSCgfp+ cells. In the early time point of ADSCgfp+ cell injection (1 day post-injection, 1 dpi), the implanted cells existed as a small cluster within the host myocardium (left, indicated by dense nuclei). Immunodetection of IL-6 revealed that, while the host cells expressed IL-6 at a weal level, the implanted cells showed strong fluorescent signal far above the autofluorescent background (right, n = 5). [file 13287_2021_2675_MOESM5_ESM.jpg]
